# Supplementary material for: Structure and tethering mechanism of dynein-2 intermediate chains in intraflagellar transport
Source: EMBO J. 2024 Mar 7;43(7):1257–72. doi: 10.1038/s44318-024-00060-1 (PMC10987677; doi:10.1038/s44318-024-00060-1)
Supplement: Supplementary file 1 — Appendix [file 44318_2024_60_MOESM1_ESM.pdf]

Structure and Tethering Mechanism of Dynein-2's Heterodimeric Intermediate Chains

Aakash G. Mukhopadhyay[1,2], Katerina Toropova[1,2], Lydia Daly[2,3], Jennifer N. Wells[2,4], Laura Vuolo[5], Miroslav Mladenov[2,6], Marian Seda[7], Dagan Jenkins[7], David J. Stephens[5], Anthony J. Roberts[1,2*]

[1]Sir William Dunn School of Pathology, University of Oxford, Oxford, UK
[2]Institute of Structural and Molecular Biology, Department of Biological Sciences, Birkbeck, University of London, London, UK
[3]Present address: Randall Centre of Cell & Molecular Biophysics, King's College London, London, UK
[4]Present address: MRC London Institute of Medical Sciences (LMS), London, UK
[5]Cell Biology Laboratories, School of Biochemistry, University of Bristol, Bristol, UK
[6]Present address: Cellular Signalling and Cytoskeletal Function Laboratory, The Francis Crick Institute, London, UK
[7]UCL Great Ormond Street Institute of Child Health, University College London, London, UK
*Correspondence: anthony.roberts@path.ox.ac.uk

APPENDIX CONTENTS

Appendix Figure S1	Page 2
Appendix Figure S2	Pages 3–4
Appendix Figure S3	Page 5
Appendix Figure S4	Page 6
Appendix Figure S5	Page 7
Appendix Table S1	Page 8
Appendix Table S2	Page 8
Appendix Table S3	Page 9

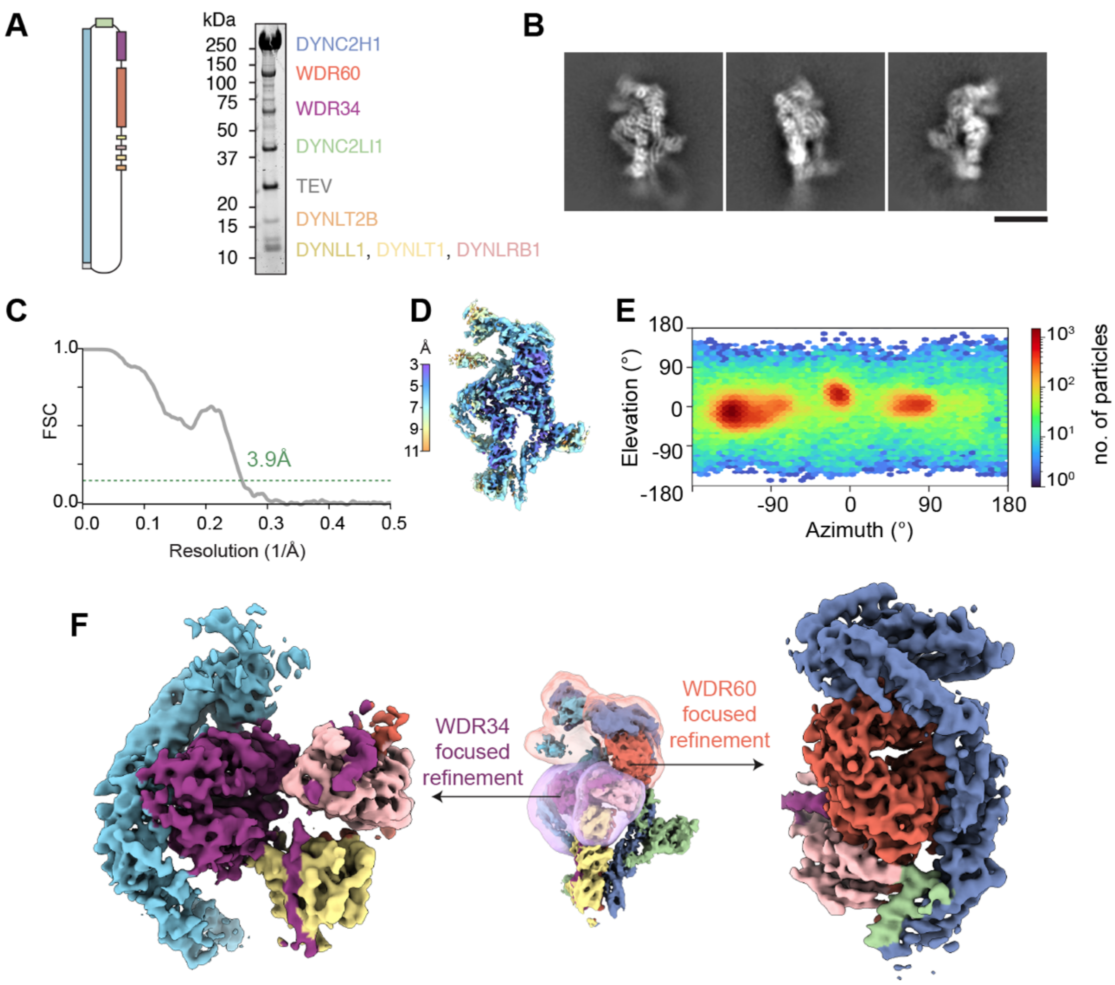

Appendix Figure S1. Cryo-EM structure determination of WDR34 and WDR60 in the dynein-2 complex

(A) Schematic of dynein-2 expression construct and SDS-PAGE of purified complex. Subunits are colored according to the code on the right.

(B) Cryo-EM class averages of dynein-2 tail domain in different orientations. Scale bar; 100 nm.

(C) Fourier shell correlation (FSC) plot for tail domain reconstruction from a consensus refinement. Global resolution at FSC=0.143 is marked.

(D) Locally sharpened reconstruction colored by local resolution according to the scale.

(E) Angular distribution of tail domain particles.

(F) Local 3D refinement of WDR34 (left panel) and WDR60 (right panel) regions in the tail domain. Locally sharpened maps, colored by subunit are shown. Middle panel; masks applied to the reference during local 3D refinement of WDR34 (transparent purple surface) and WDR60 (transparent orange surface).

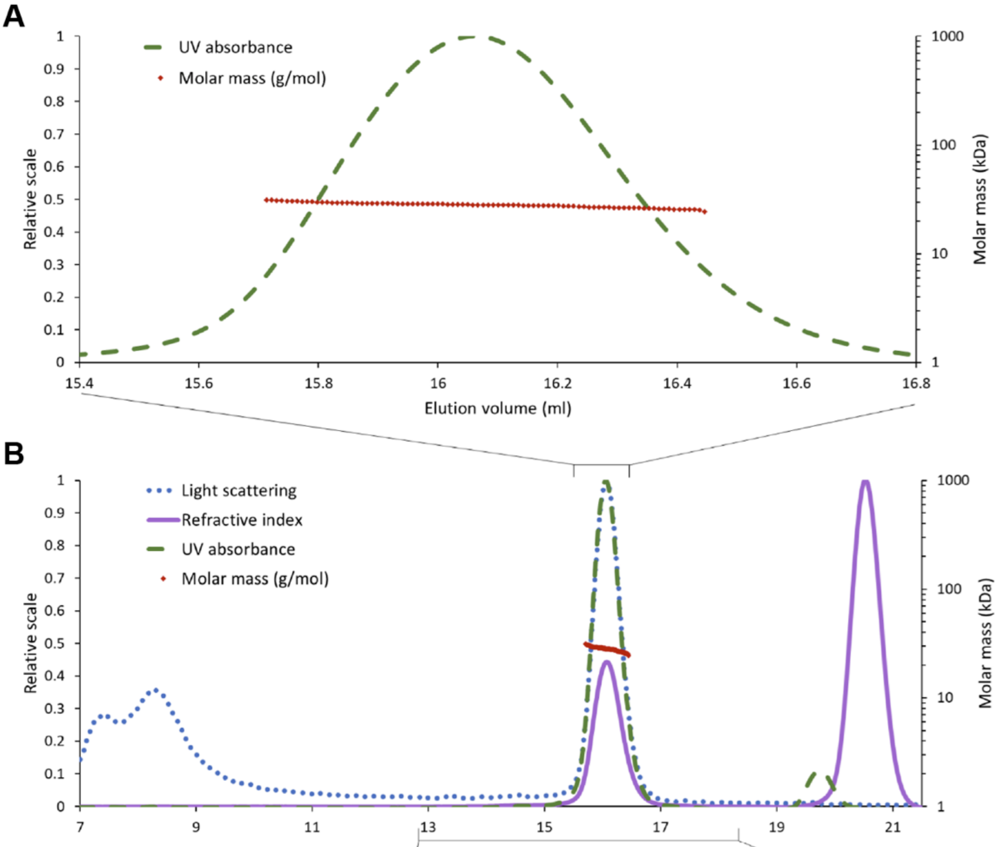

C

Mw (kDa)	28.1
Uncertainty	0.90%
Polydispersity (Mw/Mn)	1.002
Uncertainty	1.28%

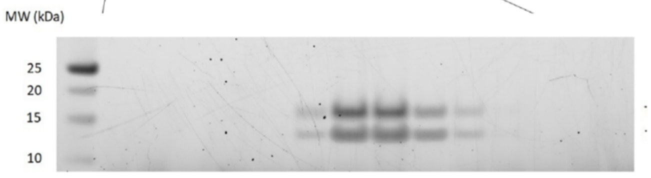

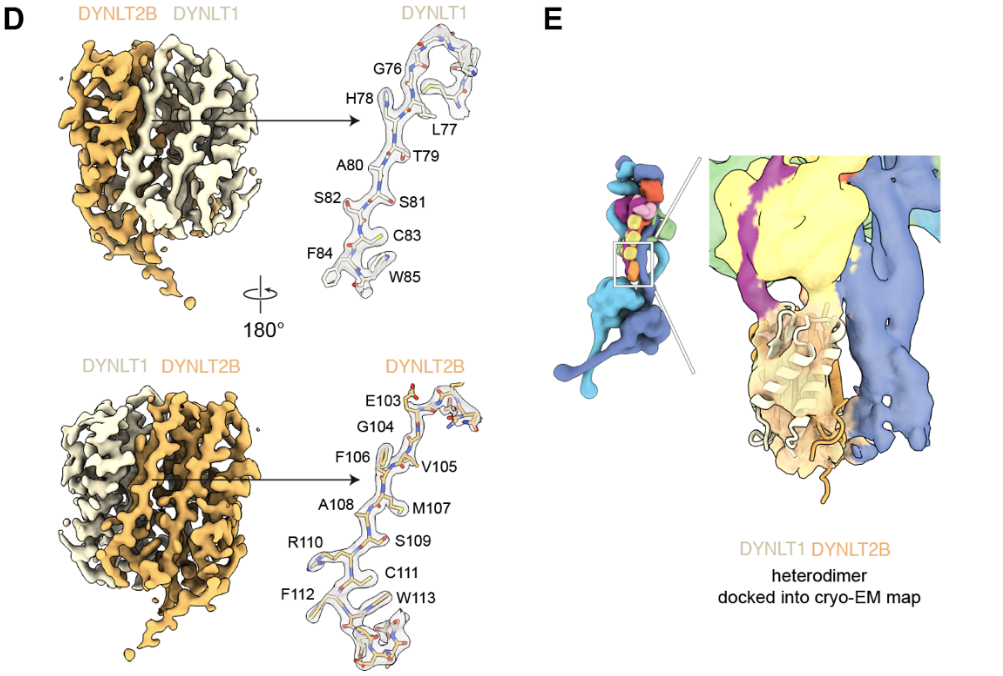

Appendix Figure S2. DYNLT1 and DYNLT2B form a heterodimer (legend continued overleaf)

Appendix Figure S2. DYNLT1 and DYNLT2B form a heterodimer (legend continued)
(A,B) SEC-MALS of the DYNLT1-DYNLT2B complex.
(**A**) Enlargement of the UV peak (green dashed line) and molar mass plot (red diamonds).
(**B**) Traces showing light scattering (blue dots), refractive index (purple line), UV absorbance (green dashed line) and molar mass (red diamonds). SDS-PAGE of the fractions across the main UV peak is shown below.
(**C**)The calculated average molar mass (28.1 kDa) matches the theoretical molar mass of a DYNLT1-DYNLT2B heterodimer (28.3 kDa).
(**D**) X-ray crystallography structure of DYNLT1-DYNLT2B. 2mFo-DFc map shown in surface representation at a threshold of 0.202. Atomic models in top and bottom panels show distinction between DYNLT1 and DYNLT2B.
(**E**) DYNLT1-DYNLT2B X-ray structure docked into the dynein-2 cryo-EM map.

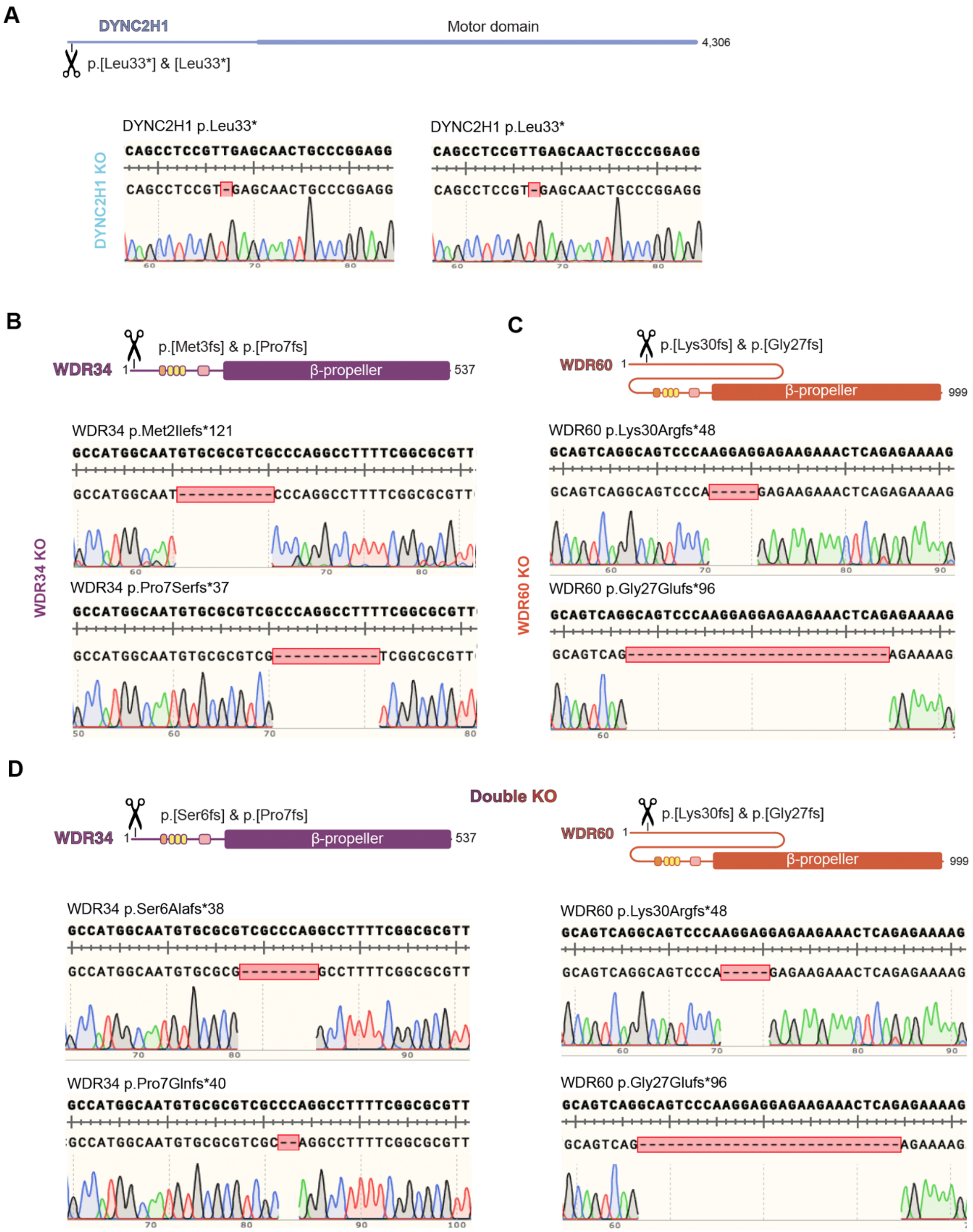

Appendix Figure S3. CRISPR knockout of dynein-2 subunits

(**A-D**) Genotype for dynein-2 subunit KO cell lines with indels highlighted by alignment with the reference sequence. Clones were exhaustively sequenced by Sanger sequencing to determine genotype (representative traces shown). DYNC2H1 KO cell line was homozygous (**A**), WDR34 KO cell line was compound heterozygous (**B**), WDR60 KO cell line was compound heterozygous (**C**), and double KO cell line was compound heterozygous for both WDR34 and WDR60 (**D**).

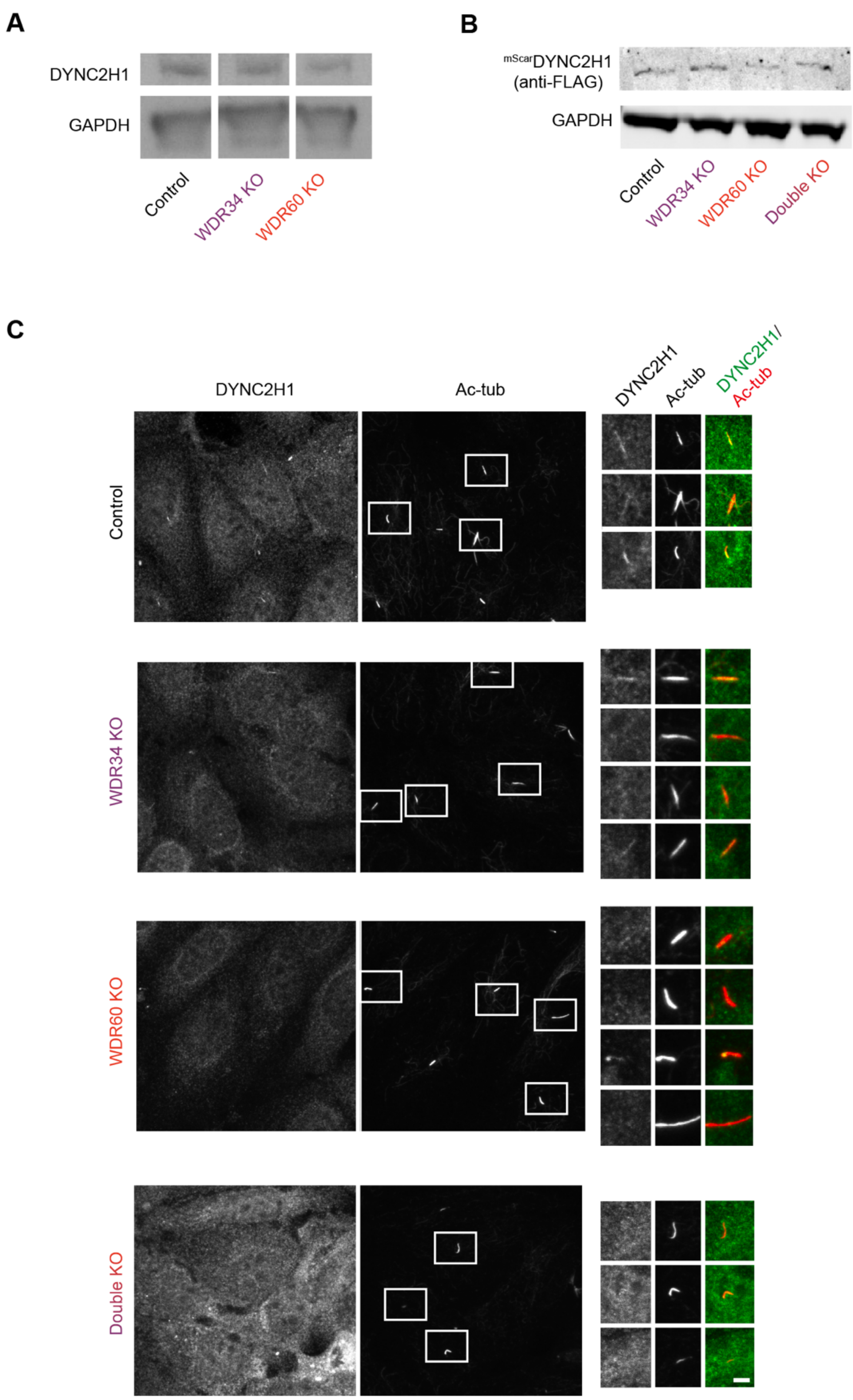

Appendix Figure S4. DYNC2H1 expression in intermediate chain KO cell lines

(**A**) Western blot showing endogenous expression levels of DYNC2H1 in indicated IMCD-3 cells. GAPDH was used as loading control.

(**B**) Western blot showing expression levels of FLAG-tagged ^{mScar}DYNC2H1 in indicated IMCD-3 cells detected using anti-FLAG. GAPDH was used as loading control.

(**C**) Immunofluorescence images probing endogenous DYNC2H1 in IMCD-3 cells lacking either or both dynein-2 intermediate chains. Scale bar 5 μm.

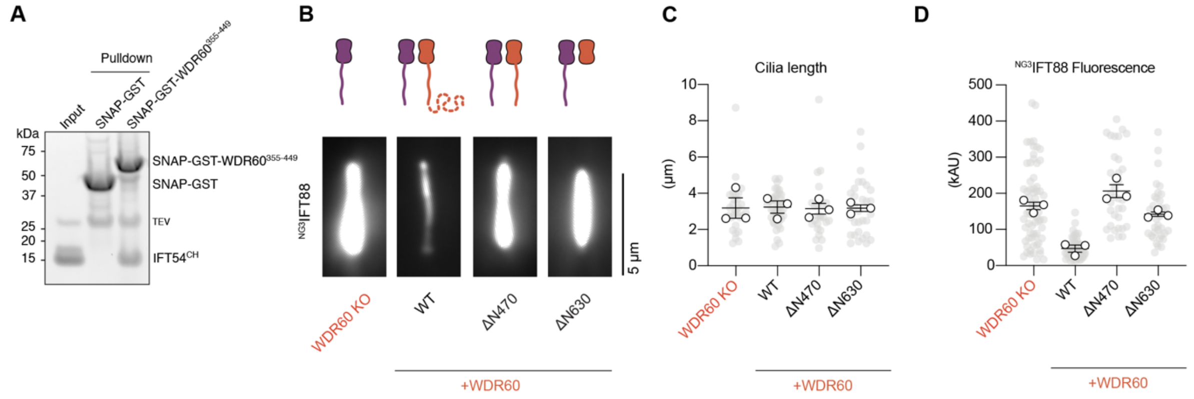

Appendix Figure S5. Analysis of the WDR60 N-terminal extension

(**A**) Pulldown experiment with purified proteins demonstrating a direct interaction between the CH domain of IFT54 ($IFT54^{CH}$) and SNAP-GST-$WDR60^{355\text{-}449}$, but not SNAP-GST.

(**B-D**) Analysis of WDR60 N-terminal truncation constructs related to Figure 5E, with constructs expressed in WDR60 KO cells rather than double KO cells.

(**B**) Representative images showing time-averaged ^{NG3}IFT88 signal in WDR60 KO cells expressing the indicated WDR60 constructs. Cartoons above images depict the corresponding intermediate chains present in the cell line and their different N-terminal truncations.

(**C**) Quantification of cilia length in WDR60 KO cells expressing the indicated WDR60 constructs from three separate experiments. Grey circles; individual data points. White circles; average from each separate experiment. Lines; mean (± SEM).

(**D**) Quantification of ^{NG3}IFT88 fluorescence intensity in WDR60 KO cells expressing the indicated WDR60 constructs from three separate experiments. 67 WDR60 KO, 49 $WDR60^{WT}$, 35 $WDR60^{\Delta N470}$ and 35 $WDR60^{\Delta N630}$ cilia were measured. Expression of $WDR60^{WT}$ rescued ^{NG3}IFT88 accumulation (one-way ANOVA followed by Kruskal-Wallis test, $p < 0.0001$). $WDR60^{\Delta N470}$ and $WDR60^{\Delta N630}$ did not rescue ^{NG3}IFT88 accumulation ($p > 0.05$).

Appendix Table S1. Cryo-EM data collection

**Data collection and processing**	
Microscope	Titan Krios (Birkbeck)
Detector	K3
Voltage (keV)	300
Nominal magnification	105,000×
Electron exposure ($e^{-}/Å^2$)	50.63
Nominal defocus range (μm)	–1.5 to –3.5
Pixel size (Å)	0.828
Particles (no.)	113,479
Global map resolution (Å)	3.9
FSC threshold	0.143
Resolution range (Å)	3.0–9.0*

*Intermediate chain regions

Appendix Table S2. WDR34 and WDR60 atomic models

	WDR34	WDR60
Initial models	AlphaFold2 Multimer: WDR34(81-536): DYNC2H1(321-771): 2 x DYNLRB1: 2 x DYNLL1:WDR60(551-607)	AlphaFold2 Multimer: WDR60(574-1057): DYNC2H1(170-771): DYNC2LI1(308-343): 2 x DYNLRB1
Model composition		
Chains	B,C,D,G,H,I,J	A,C,D,E,G,H
Nonhydrogen atoms	7,680	9,687
Protein residues	1,156	1,287
R.m.s. deviations		
Bond lengths (Å)	0.012	0.011
Bond angles (°)	1.928	1.987
FSC model (0.143 / 0.5)	4.0 / 8.1	3.9 / 4.2
Correlation coefficient (CCmask / box)	0.46 / 0.70	0.66 / 0.82
**Validation**		
MolProbity score	0.84	0.98
Clashscore	0.14	0.43
Poor rotamers (%)	0	0
Ramachandran plot		
Favored (%)	95.87	95.34
Allowed (%)	4.13	4.66
Disallowed (%)	0	0

Residues numbers used in AlphaFold2 Multimer analysis in parentheses.

Appendix Table S3: X-ray data collection and refinement statistics for TCTEX1D2-DYNLT1

**Data Collection**	
Wavelength (Å)	0.98
Resolution range (Å)	45.68–2.02
Space group	P 41 21 2
Cell dimensions	
a, *b*, *c* (Å)	78.15, 78.15, 112.56
α, β, γ (°)	90, 90, 90
Total reflections	147134 (22107)
Unique reflections	24180 (3754)
Redundancy	6.08 (5.89)
Completeness (%)	99.5% (97.7%)
I / σI	14.56 (0.65)
Wilson B-factor (Å^2)	60.5
R-meas (%)	5.6 (263.8)
CC1/2	99.9 (26.5)
**Refinement**	
Resolution (Å)	2.02
R-work/R-free (%)	0.270 / 0.259
No. atoms	1830
Average B-factor (Å^2)	72.0
R.m.s. deviations	
Bond lengths (Å)	0.007
Bond angles (°)	0.58
**Ramachandran plot**	
Favored (%)	98.68
Allowed (%)	1.32
Outliers (%)	0.00
Clashscore	2.21

Values in parentheses are for highest-resolution shell (2.12 – 2.00).
