# Supplementary figures and images for: Structure and tethering mechanism of dynein-2 intermediate chains in intraflagellar transport
Source: EMBO J. 2024 Mar 7;43(7):1257–72. doi: 10.1038/s44318-024-00060-1 (PMC10987677; doi:10.1038/s44318-024-00060-1)

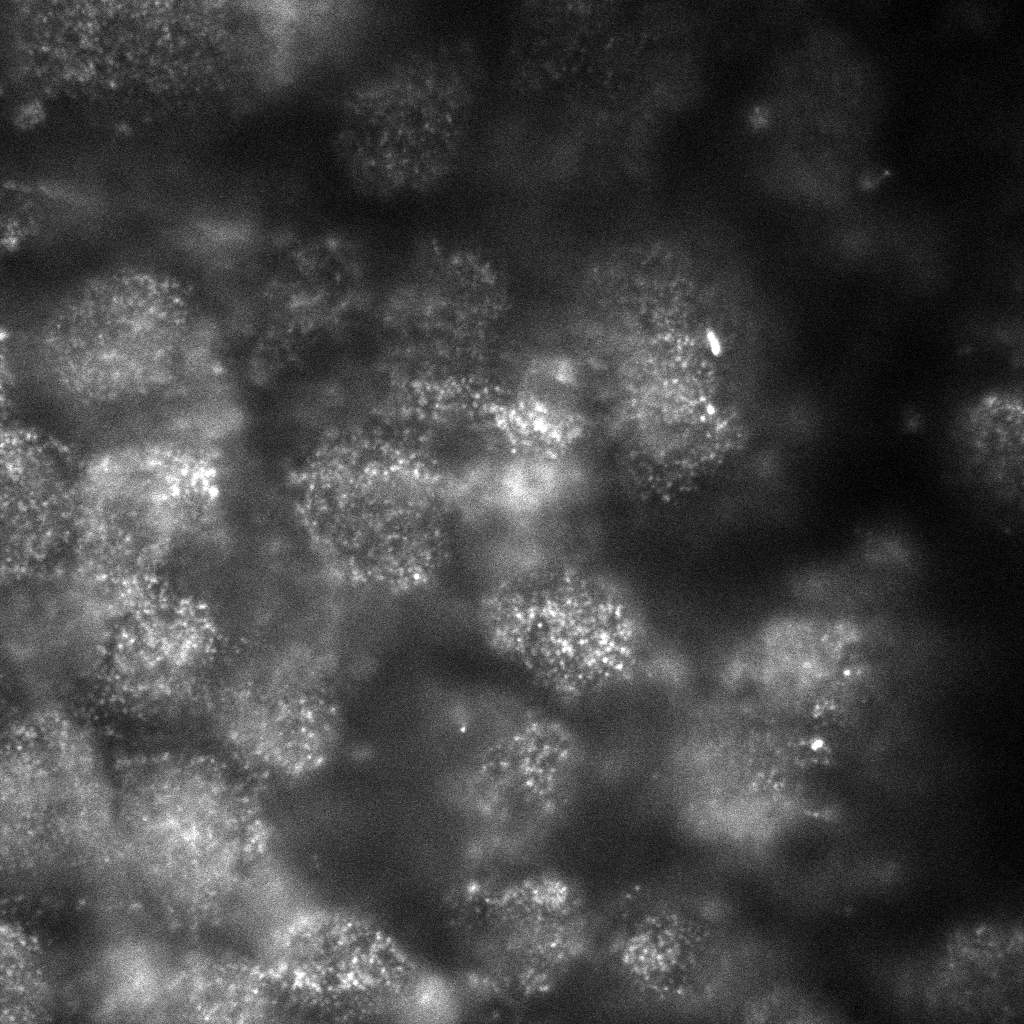

Supplement: Supplementary file 3 — Source Data Fig. 2 [file 44318_2024_60_MOESM3_ESM.zip › Figure 2/2F/Double KO +SAG Smo.tif]

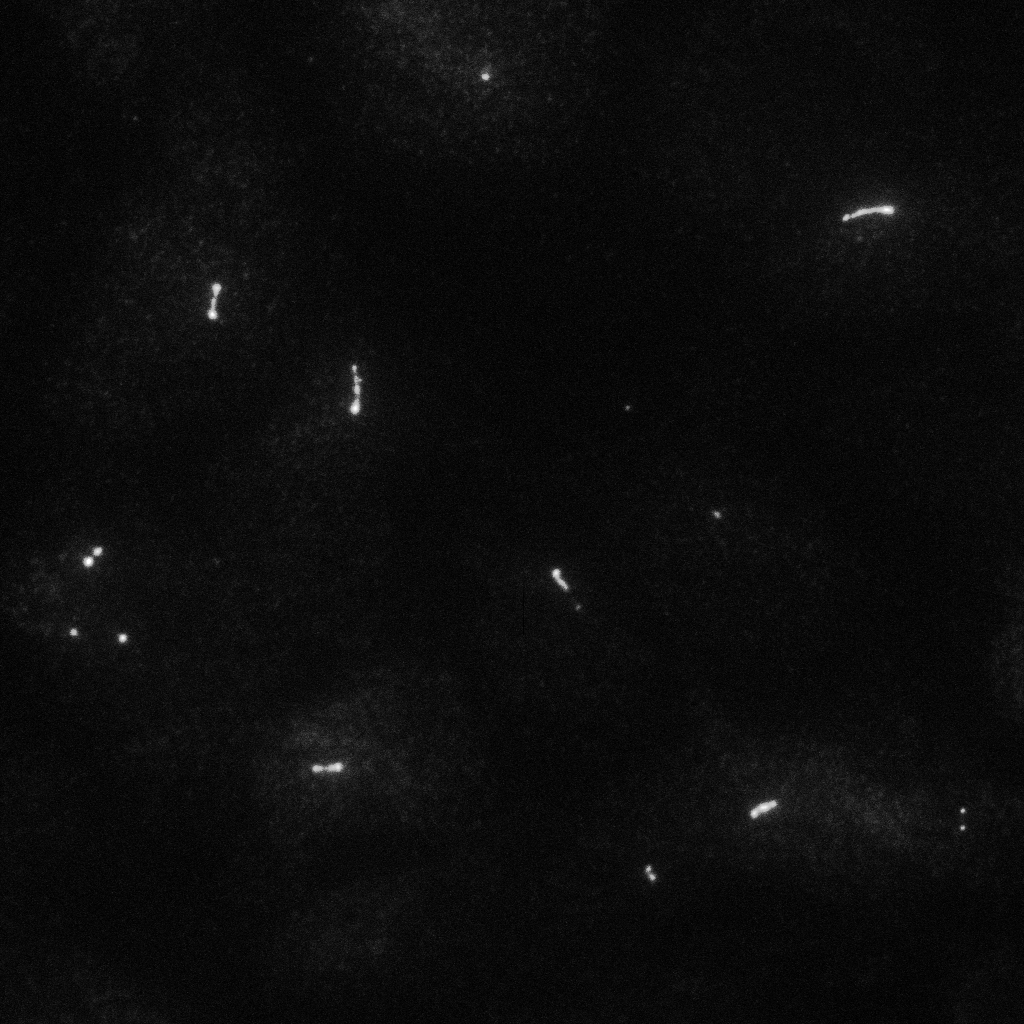

Supplement: Supplementary file 3 — Source Data Fig. 2 [file 44318_2024_60_MOESM3_ESM.zip › Figure 2/2F/WDR34 KO +SAG NG3IFT88.tif]

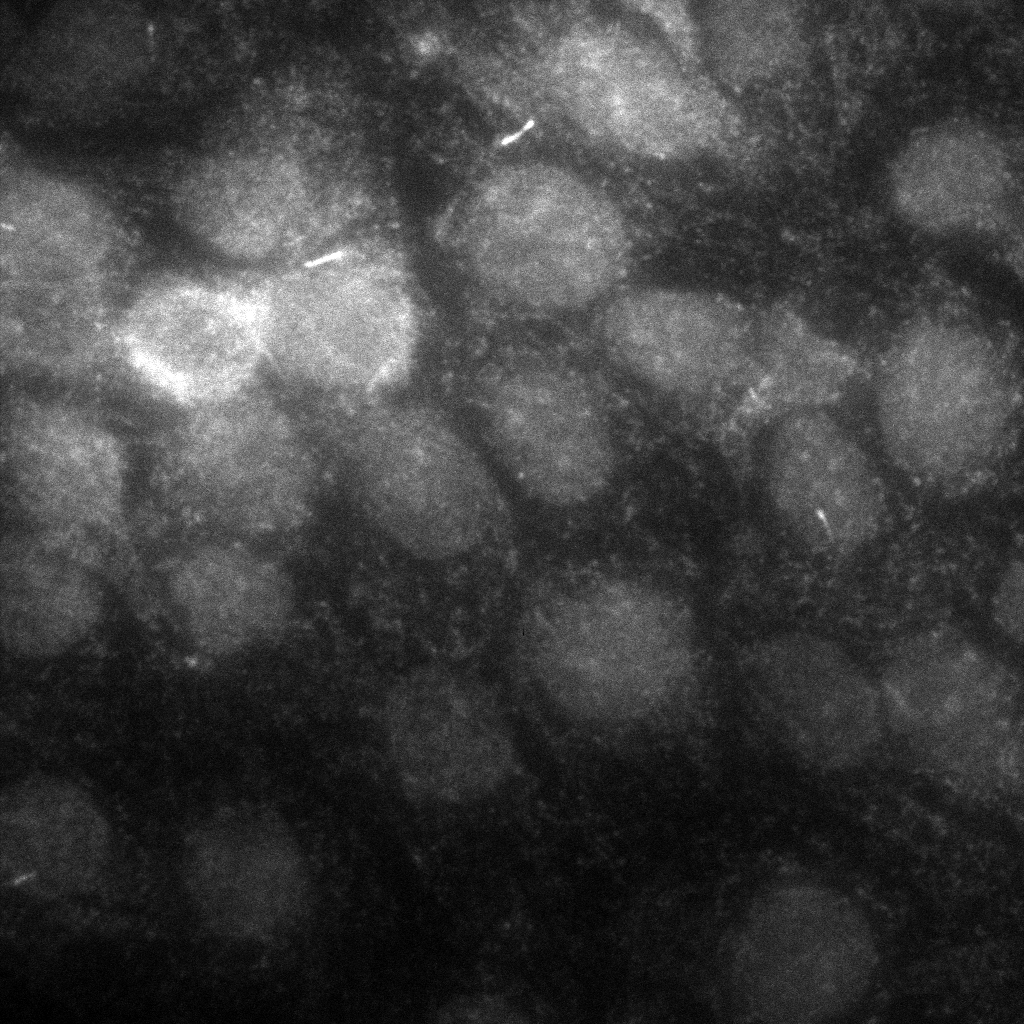

Supplement: Supplementary file 3 — Source Data Fig. 2 [file 44318_2024_60_MOESM3_ESM.zip › Figure 2/2F/WDR60 KO +SAG Smo.tif]

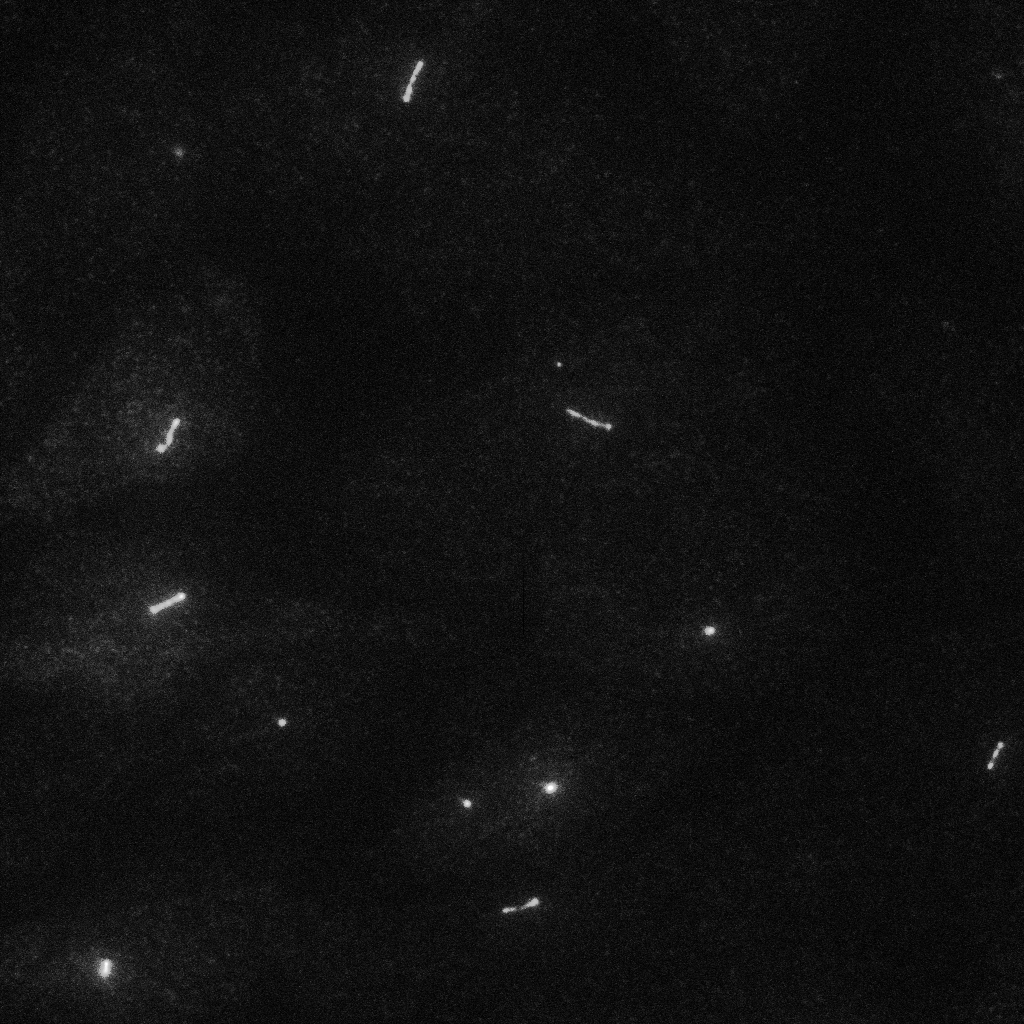

Supplement: Supplementary file 3 — Source Data Fig. 2 [file 44318_2024_60_MOESM3_ESM.zip › Figure 2/2F/WDR34 KO -SAG NG3IFT88.tif]

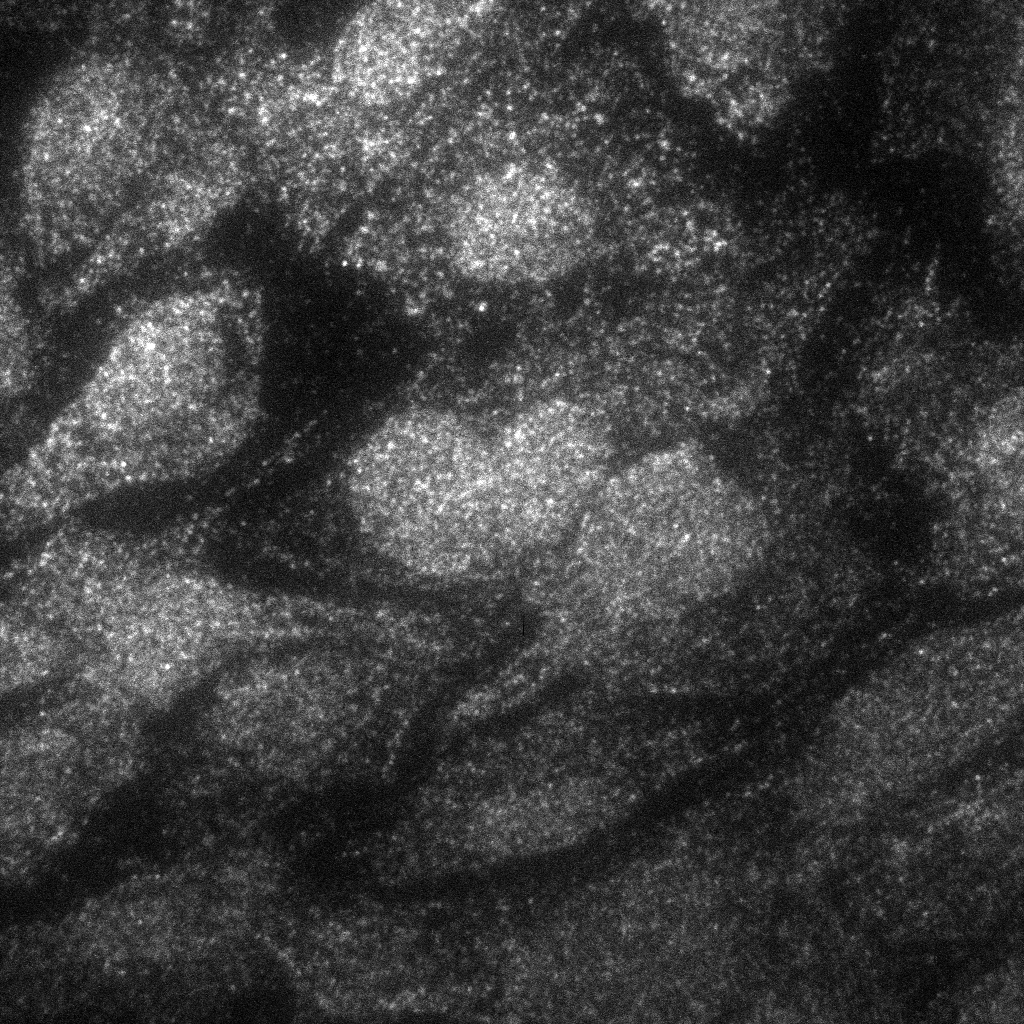

Supplement: Supplementary file 3 — Source Data Fig. 2 [file 44318_2024_60_MOESM3_ESM.zip › Figure 2/2F/WDR34 KO -SAG Smo.tif]

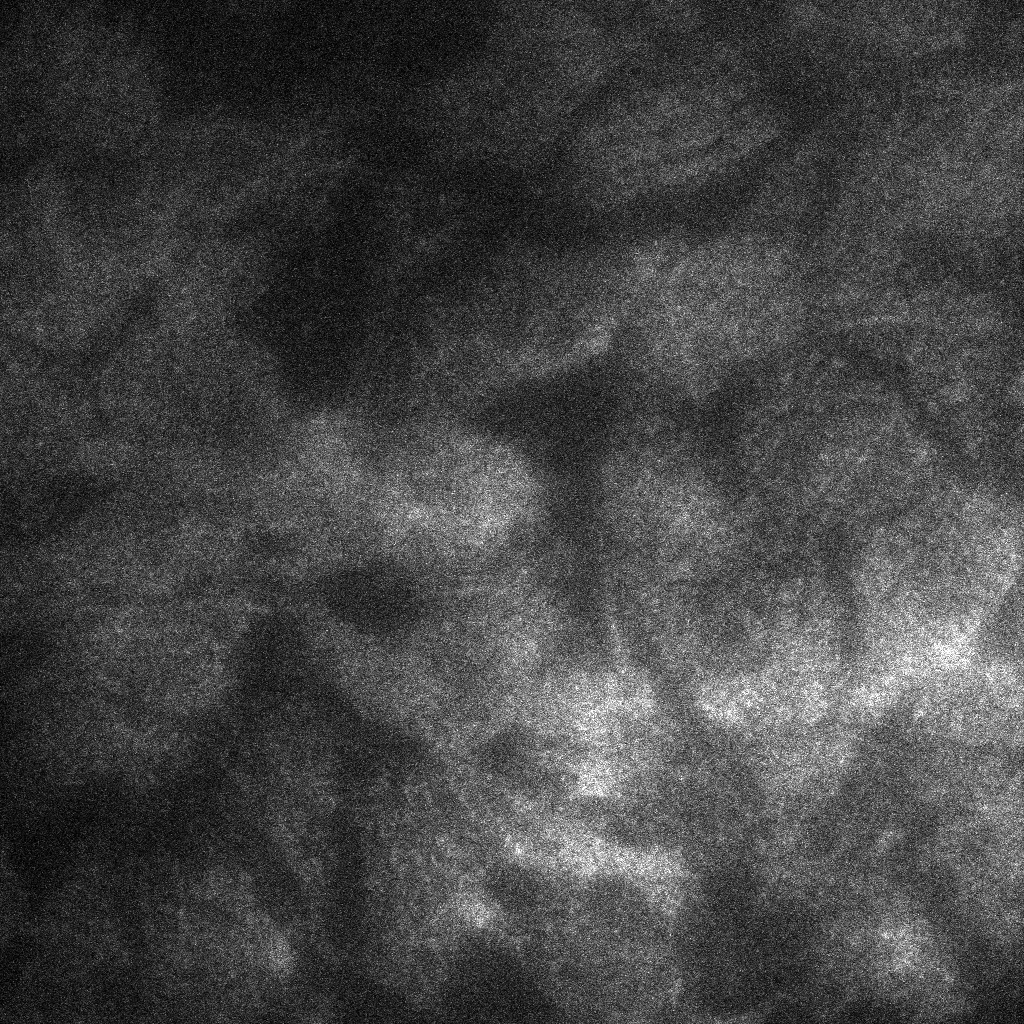

Supplement: Supplementary file 3 — Source Data Fig. 2 [file 44318_2024_60_MOESM3_ESM.zip › Figure 2/2F/Control -SAG Smo.tif]

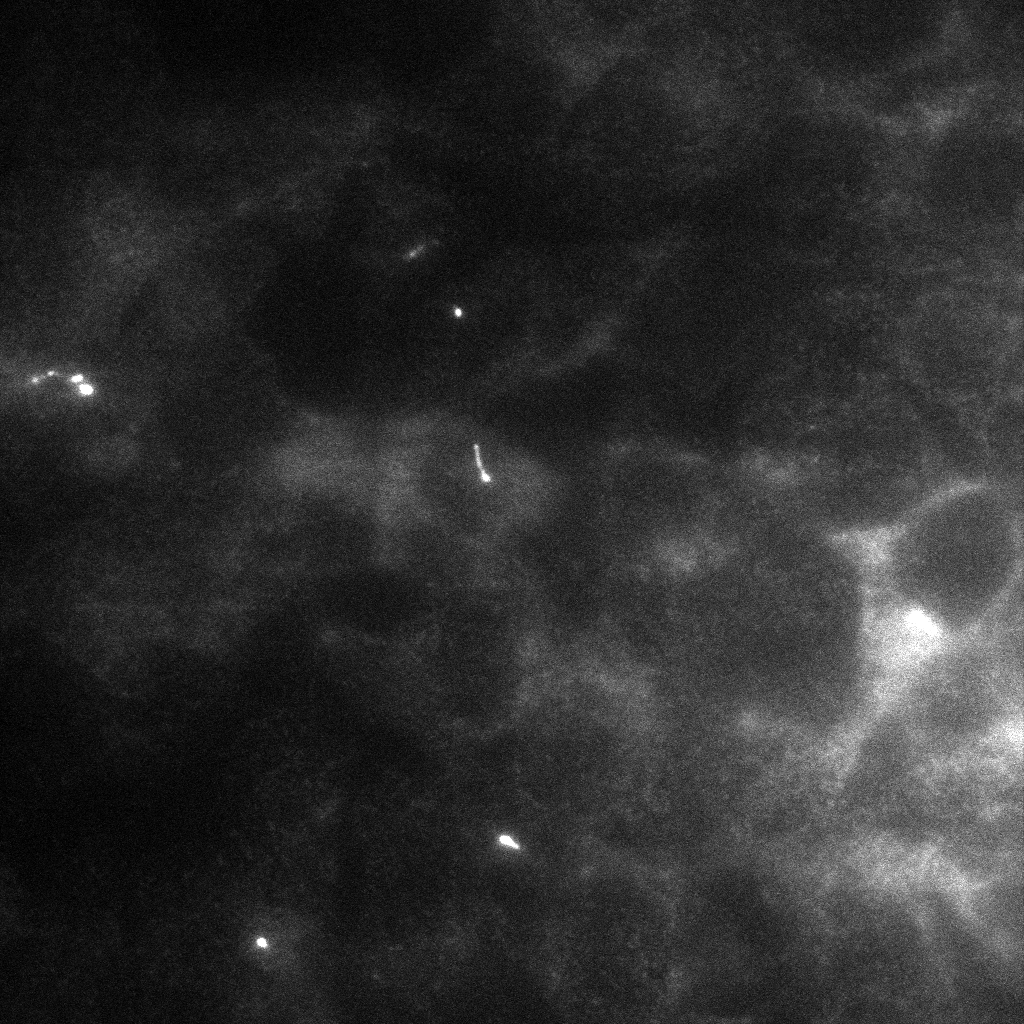

Supplement: Supplementary file 3 — Source Data Fig. 2 [file 44318_2024_60_MOESM3_ESM.zip › Figure 2/2F/Control -SAG NG3IFT88.tif]

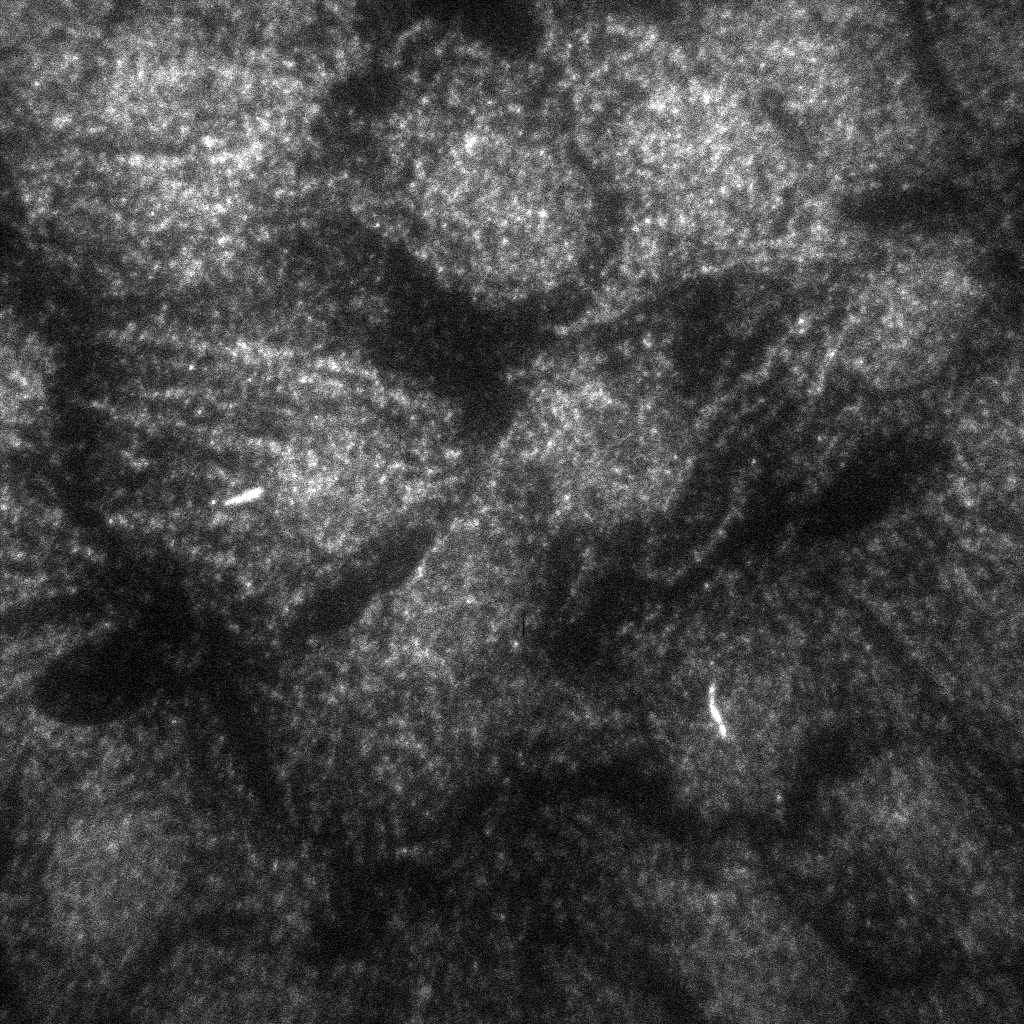

Supplement: Supplementary file 3 — Source Data Fig. 2 [file 44318_2024_60_MOESM3_ESM.zip › Figure 2/2F/WDR60 KO -SAG Smo.tif]

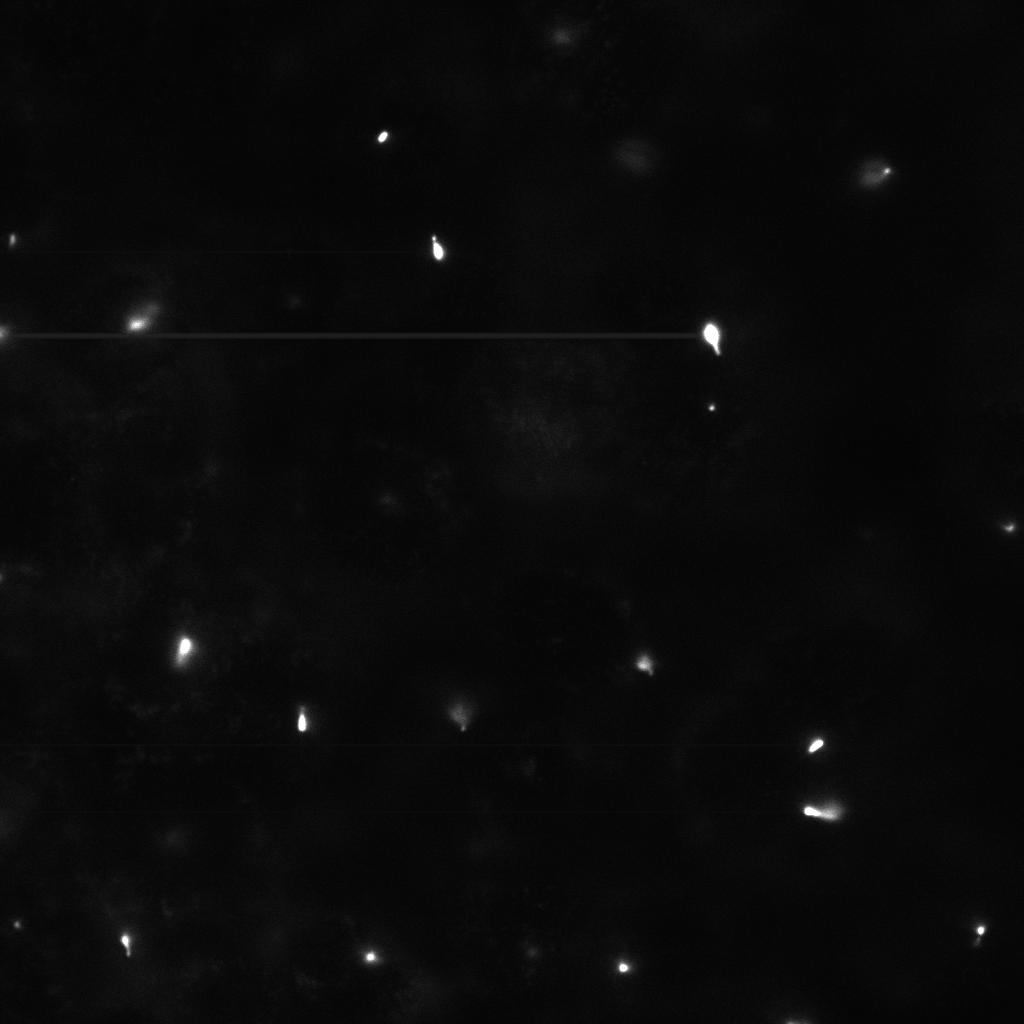

Supplement: Supplementary file 3 — Source Data Fig. 2 [file 44318_2024_60_MOESM3_ESM.zip › Figure 2/2F/Double KO +SAG NG3IFT88.tif]

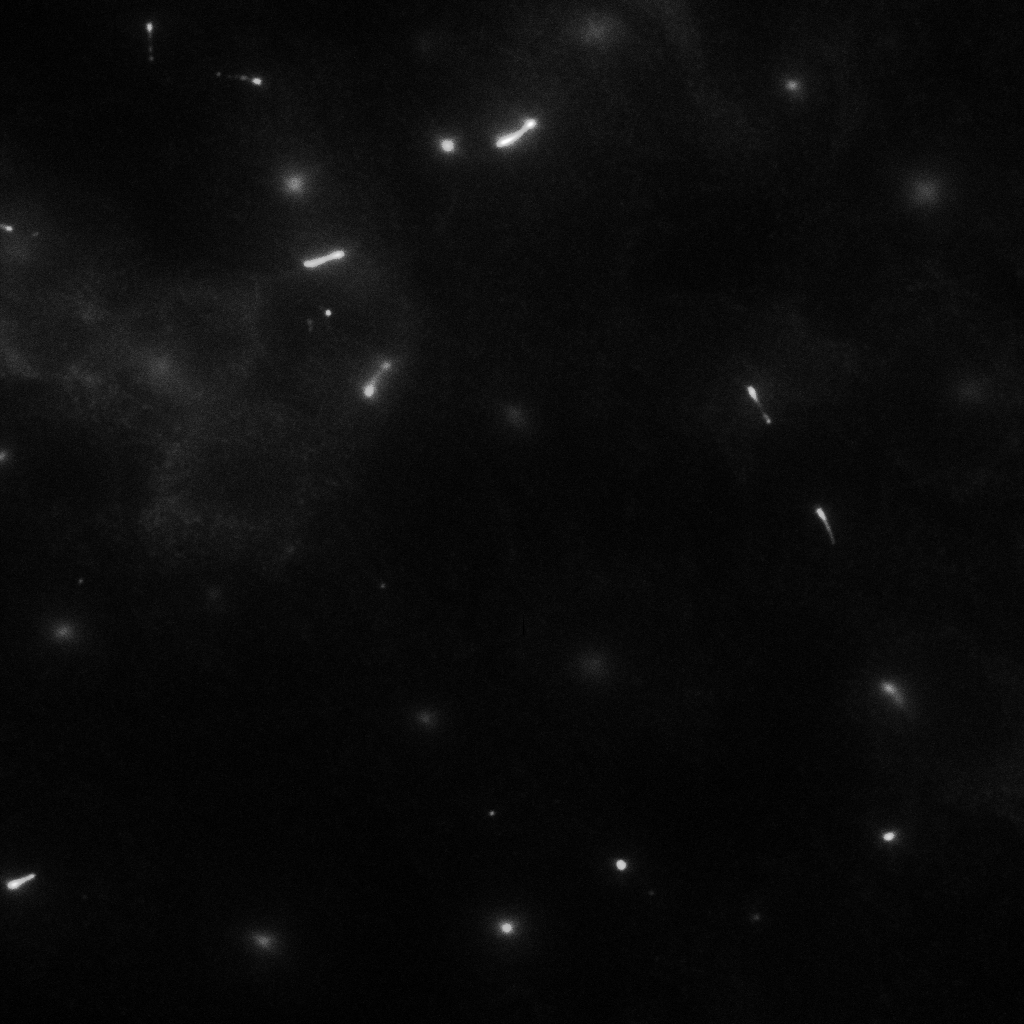

Supplement: Supplementary file 3 — Source Data Fig. 2 [file 44318_2024_60_MOESM3_ESM.zip › Figure 2/2F/WDR60 KO +SAG NG3IFT88.tif]

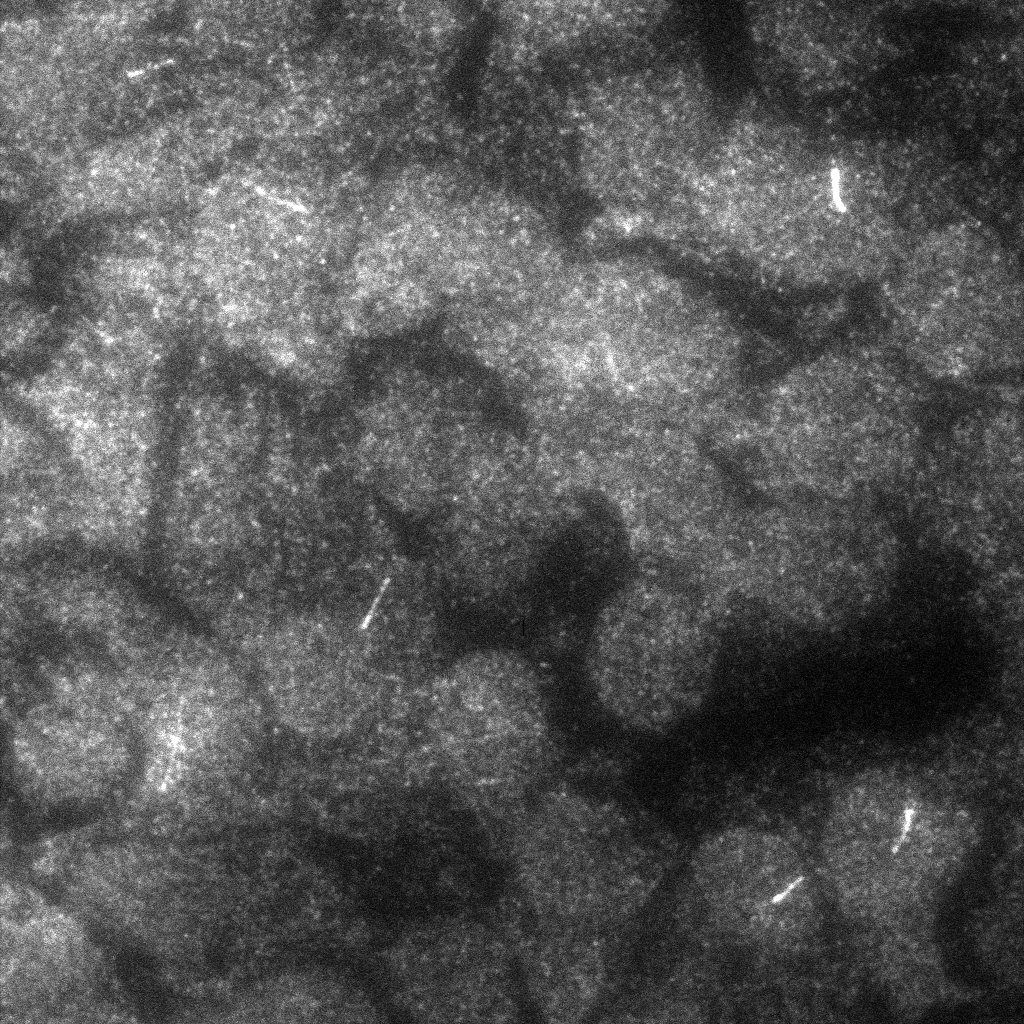

Supplement: Supplementary file 3 — Source Data Fig. 2 [file 44318_2024_60_MOESM3_ESM.zip › Figure 2/2F/Control +SAG Smo.tif]

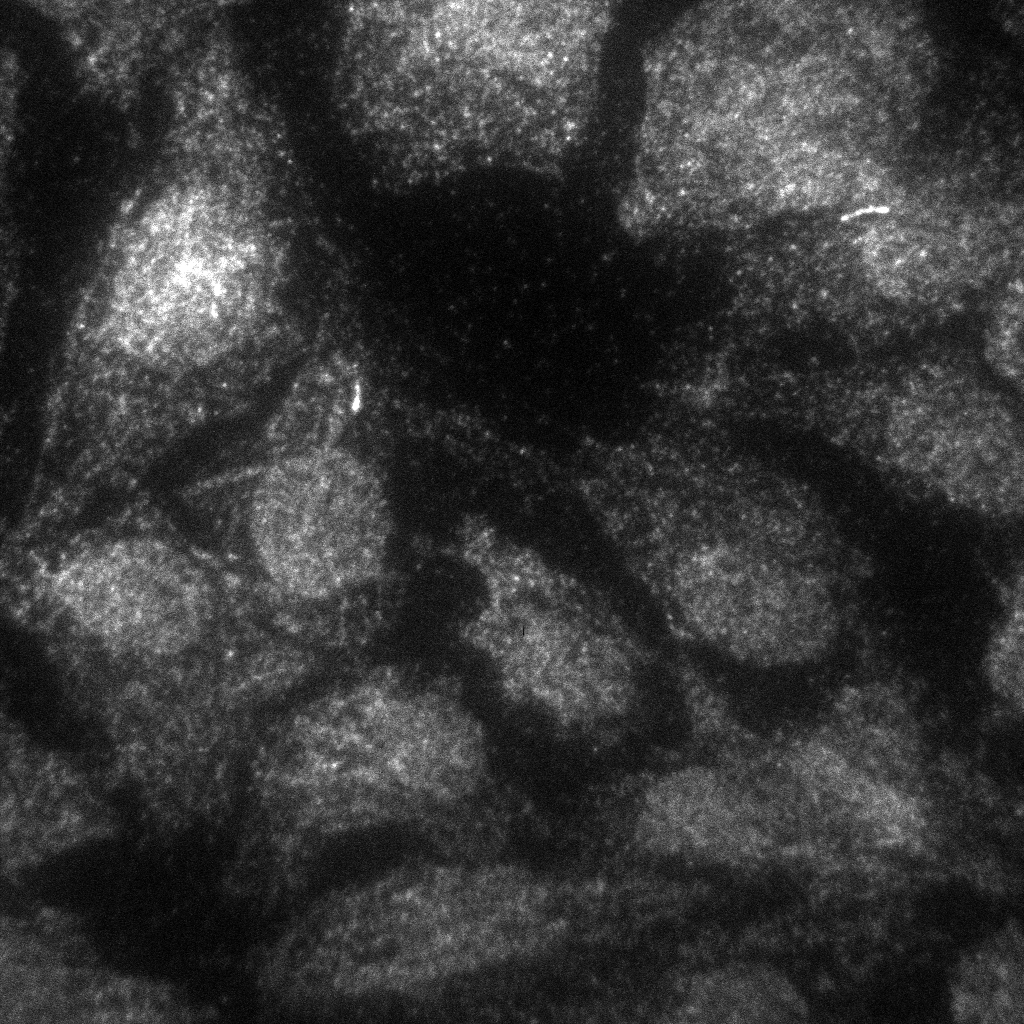

Supplement: Supplementary file 3 — Source Data Fig. 2 [file 44318_2024_60_MOESM3_ESM.zip › Figure 2/2F/WDR34 KO +SAG Smo.tif]

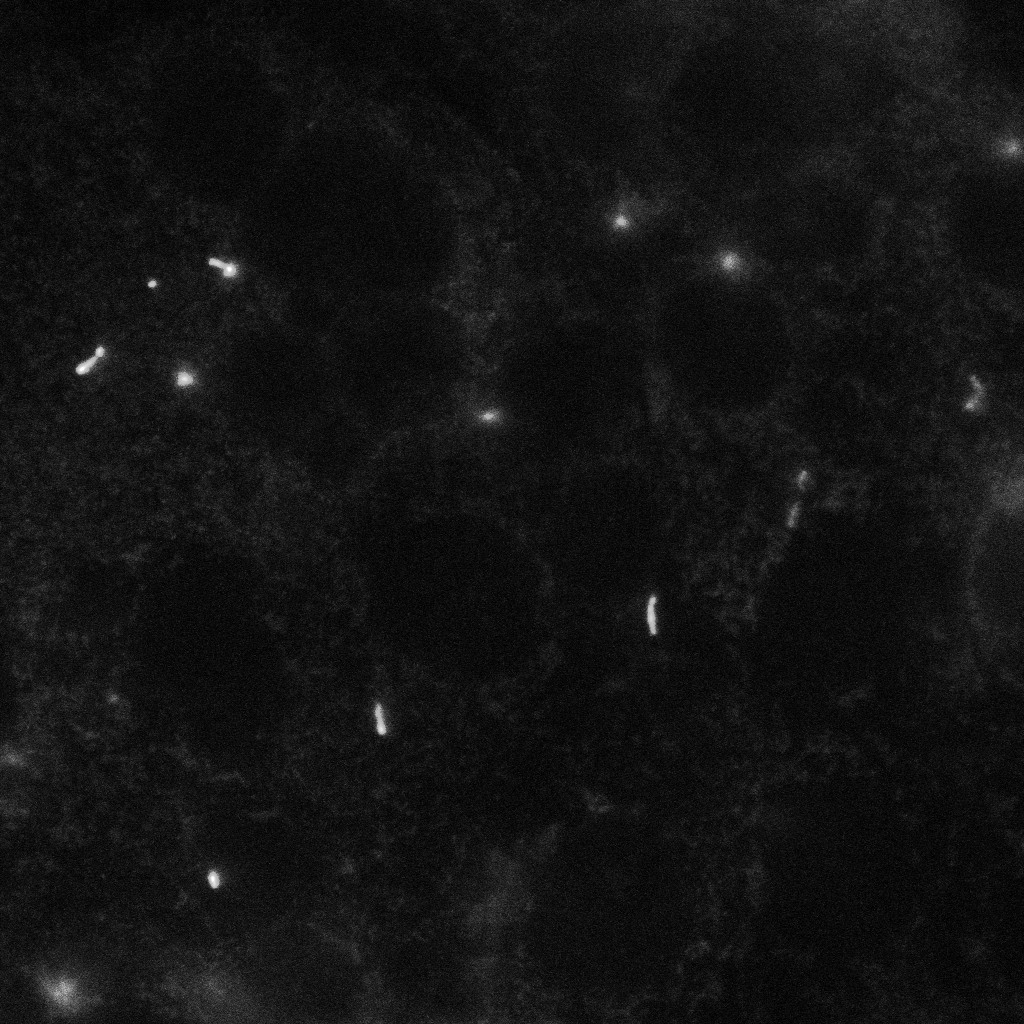

Supplement: Supplementary file 3 — Source Data Fig. 2 [file 44318_2024_60_MOESM3_ESM.zip › Figure 2/2F/Double KO -SAG NG3IFT88.tif]

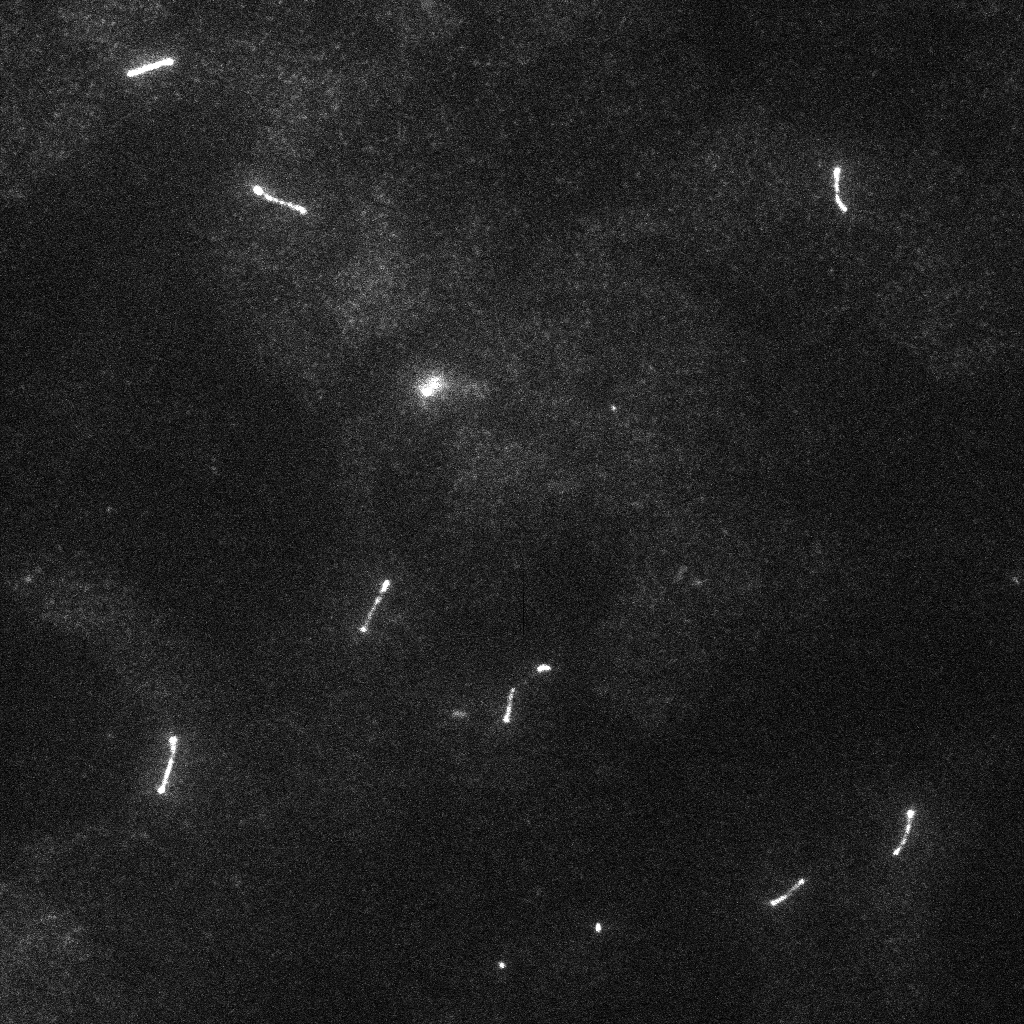

Supplement: Supplementary file 3 — Source Data Fig. 2 [file 44318_2024_60_MOESM3_ESM.zip › Figure 2/2F/Control +SAG NG3IFT88.tif]

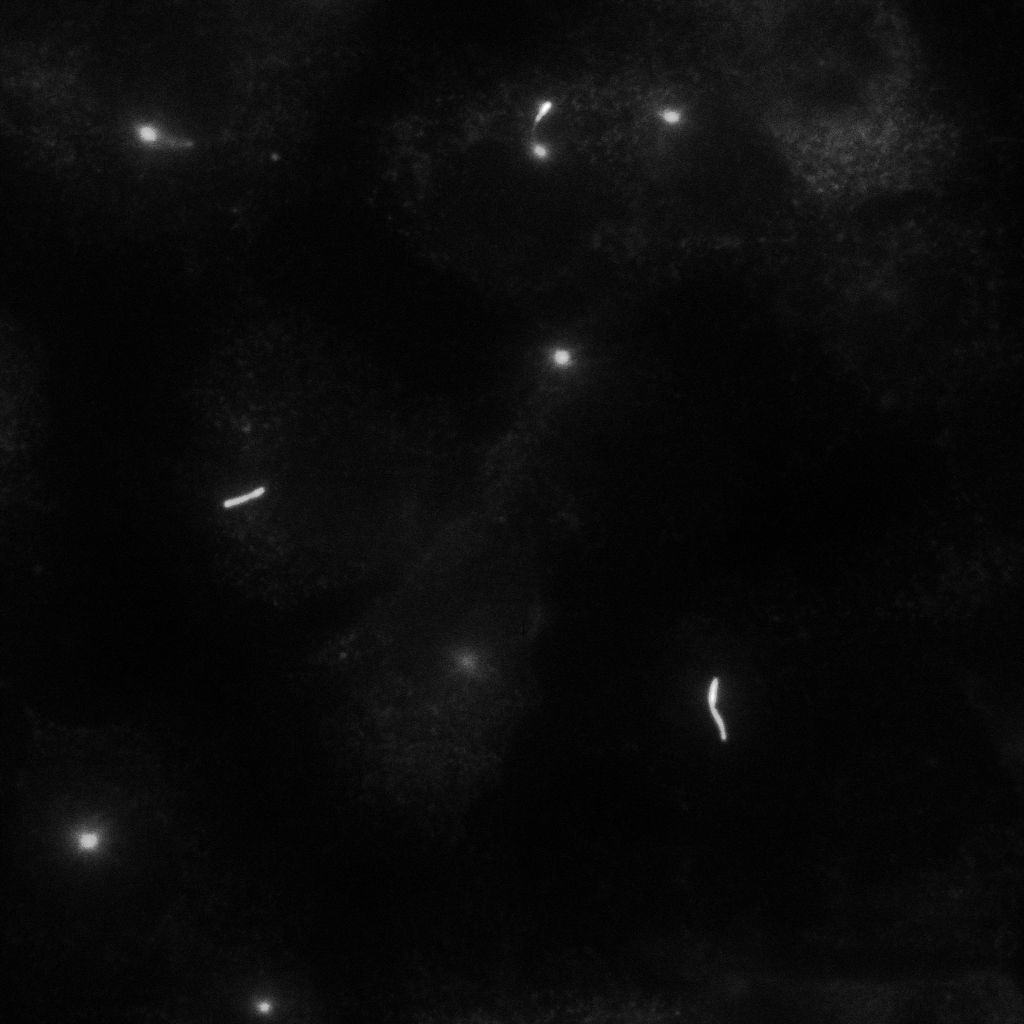

Supplement: Supplementary file 3 — Source Data Fig. 2 [file 44318_2024_60_MOESM3_ESM.zip › Figure 2/2F/WDR60 KO -SAG NG3IFT88.tif]

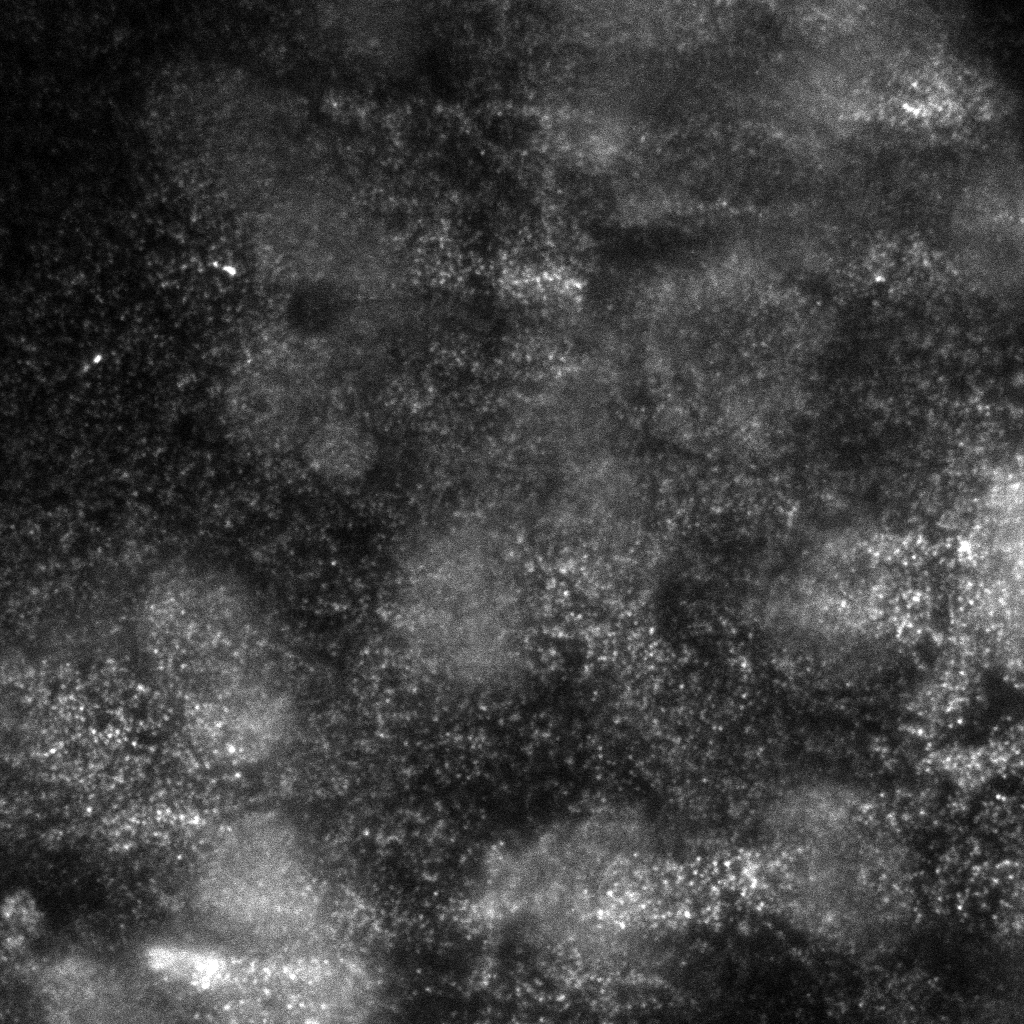

Supplement: Supplementary file 3 — Source Data Fig. 2 [file 44318_2024_60_MOESM3_ESM.zip › Figure 2/2F/Double KO -SAG Smo.tif]

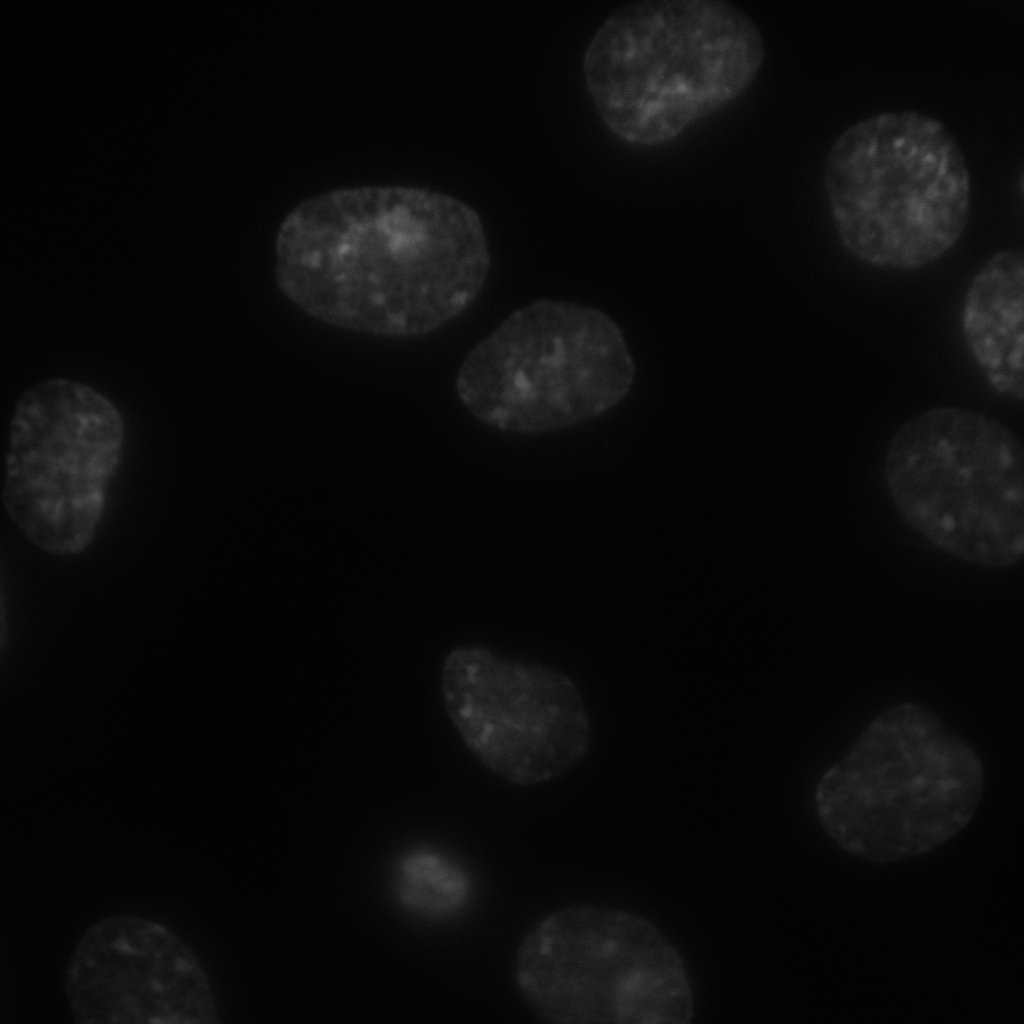

Supplement: Supplementary file 3 — Source Data Fig. 2 [file 44318_2024_60_MOESM3_ESM.zip › Figure 2/2C/Control.tif]

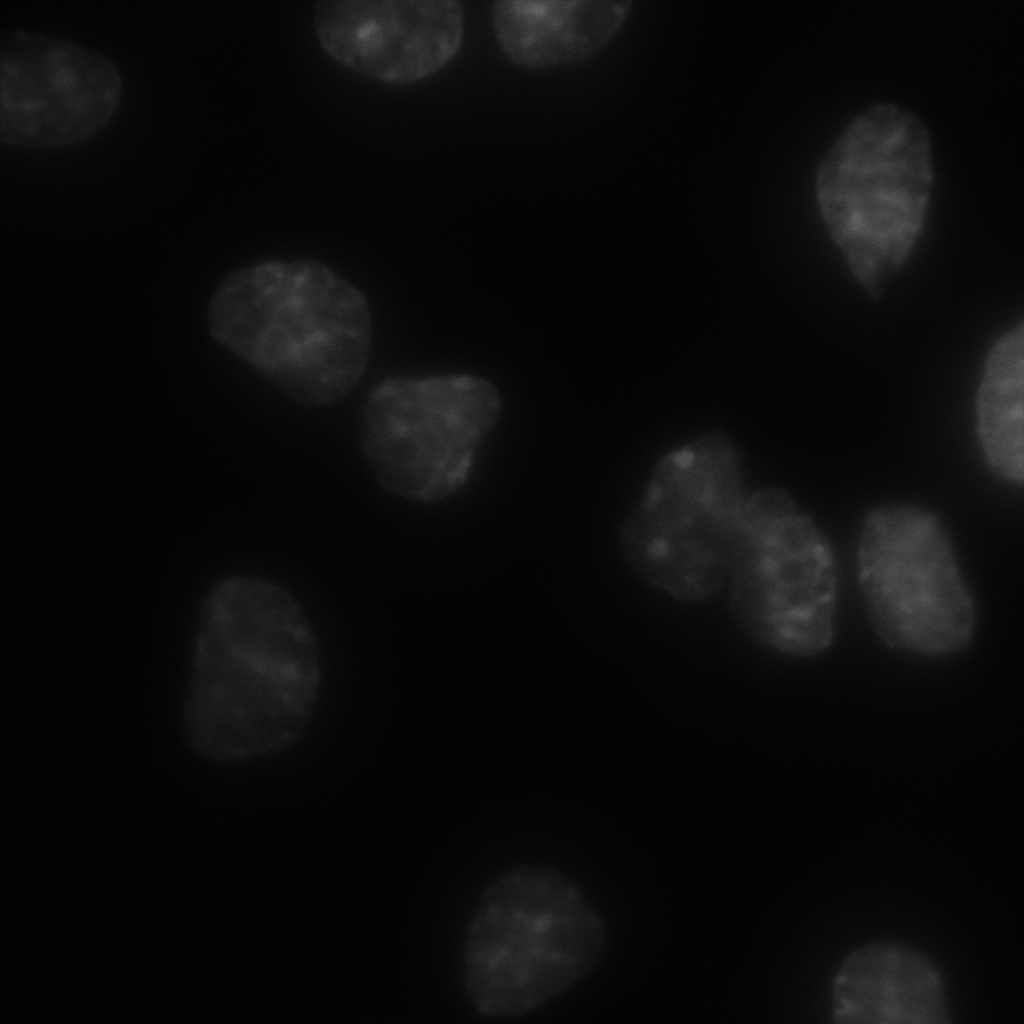

Supplement: Supplementary file 3 — Source Data Fig. 2 [file 44318_2024_60_MOESM3_ESM.zip › Figure 2/2C/DYNC2H1 KO.tif]

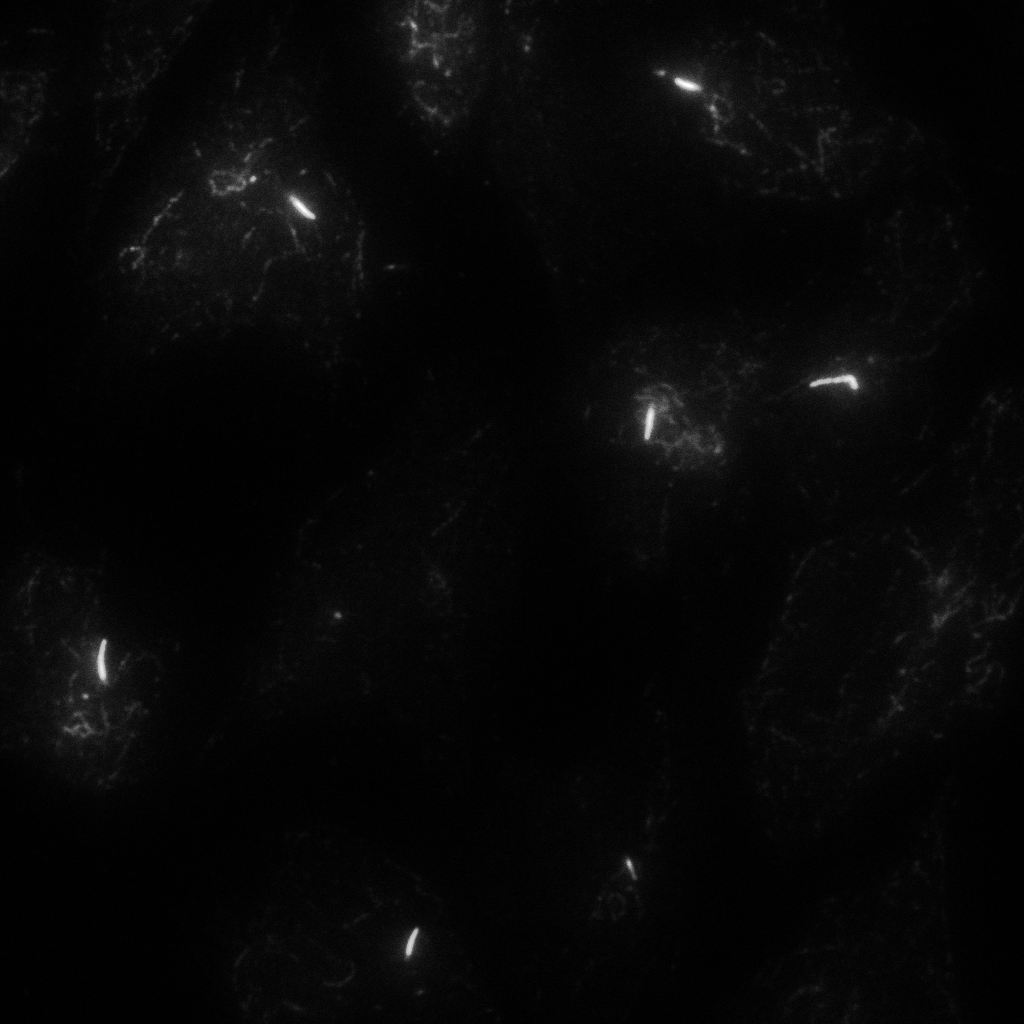

Supplement: Supplementary file 3 — Source Data Fig. 2 [file 44318_2024_60_MOESM3_ESM.zip › Figure 2/2C/WDR34 KO.tif]

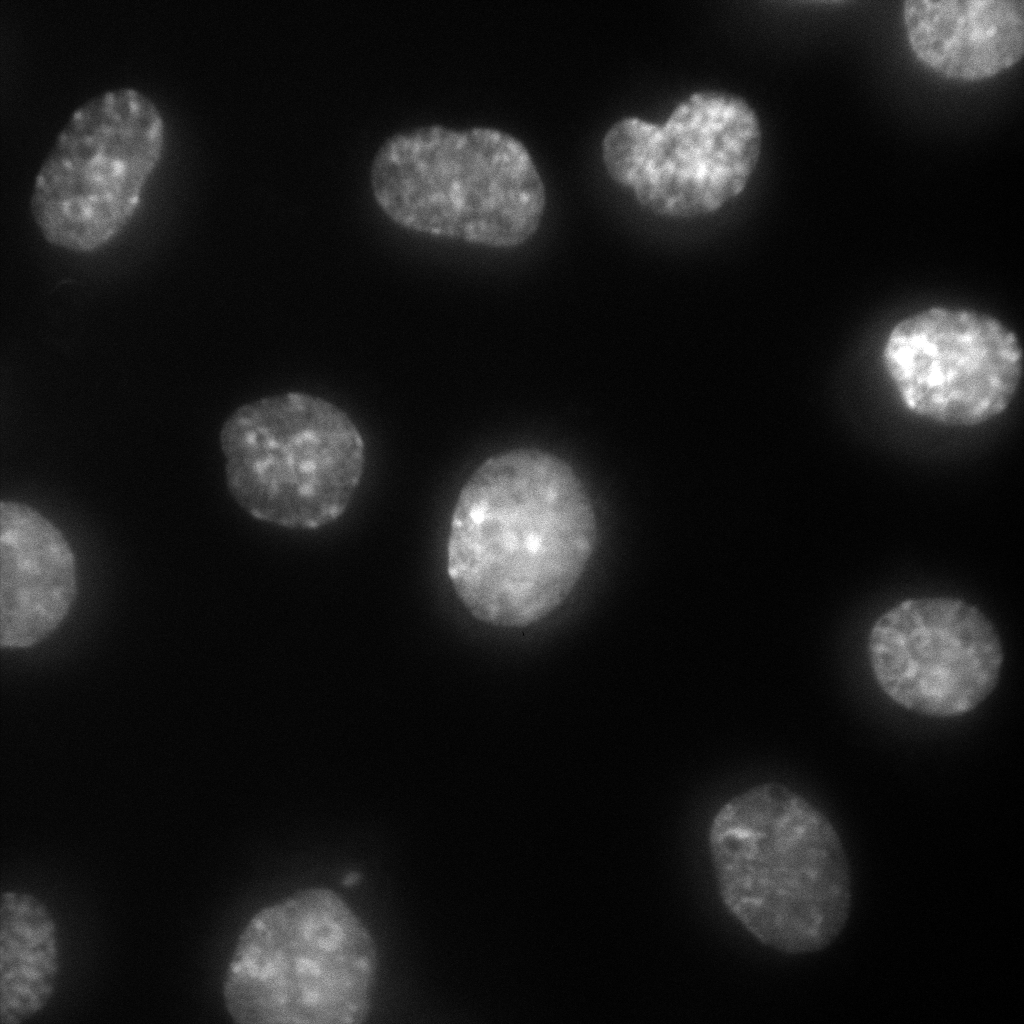

Supplement: Supplementary file 3 — Source Data Fig. 2 [file 44318_2024_60_MOESM3_ESM.zip › Figure 2/2C/WDR60 KO.tif]

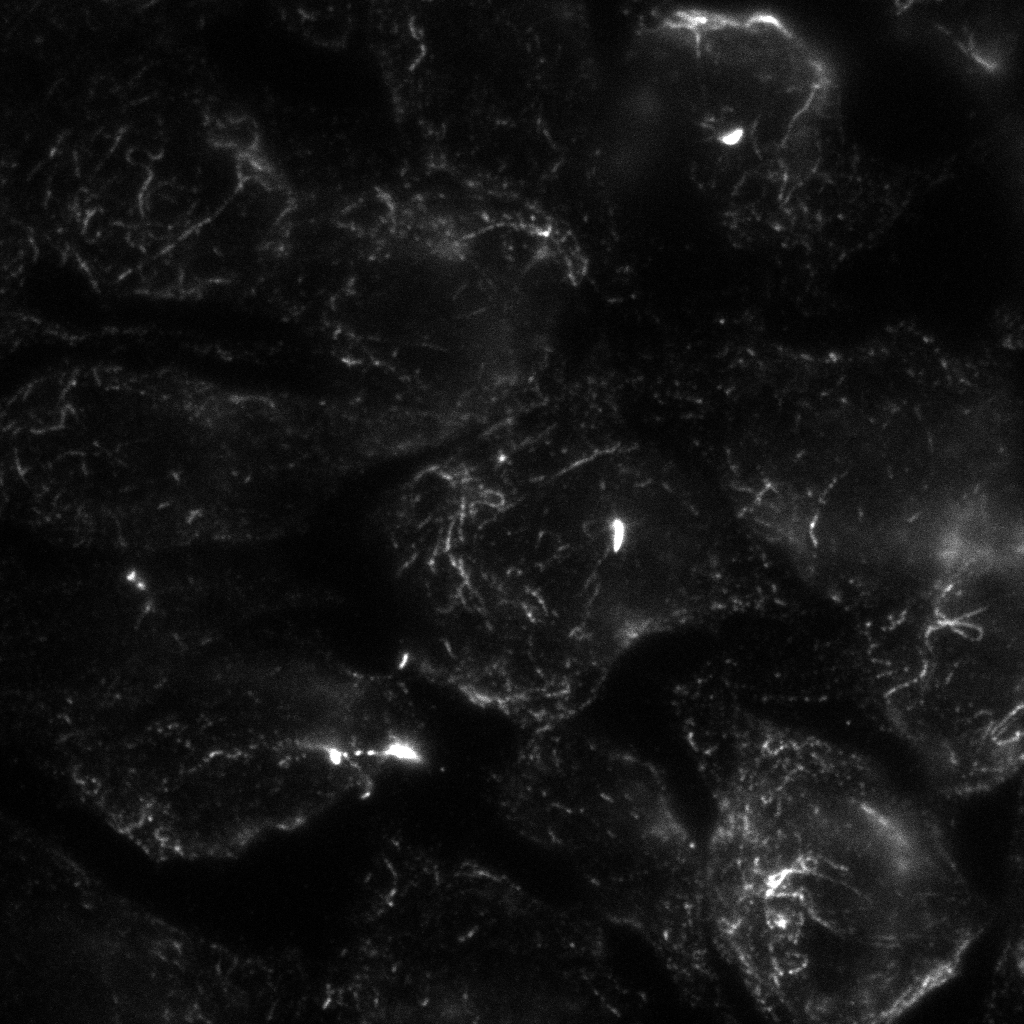

Supplement: Supplementary file 3 — Source Data Fig. 2 [file 44318_2024_60_MOESM3_ESM.zip › Figure 2/2C/Double KO.tif]

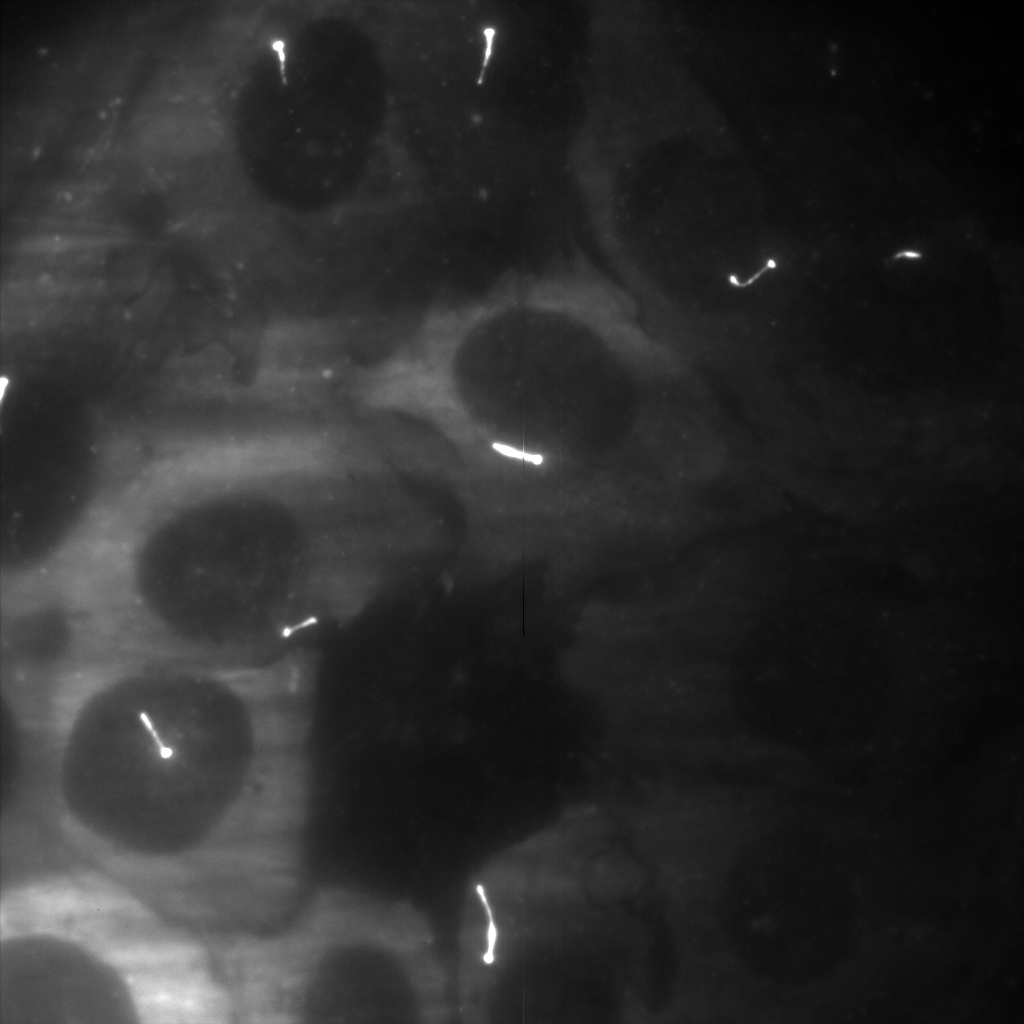

Supplement: Supplementary file 3 — Source Data Fig. 2 [file 44318_2024_60_MOESM3_ESM.zip › Figure 2/2D/Control.tif]

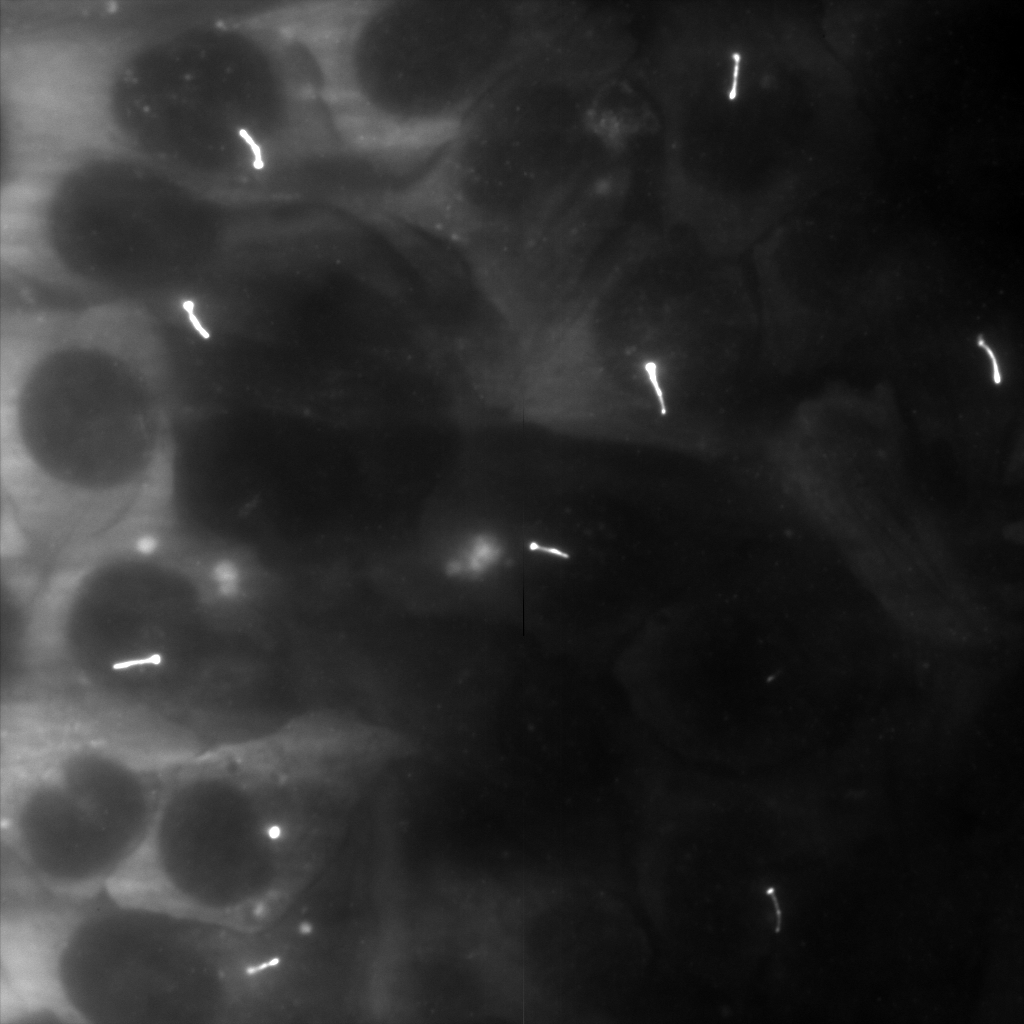

Supplement: Supplementary file 3 — Source Data Fig. 2 [file 44318_2024_60_MOESM3_ESM.zip › Figure 2/2D/WDR34 KO.tif]

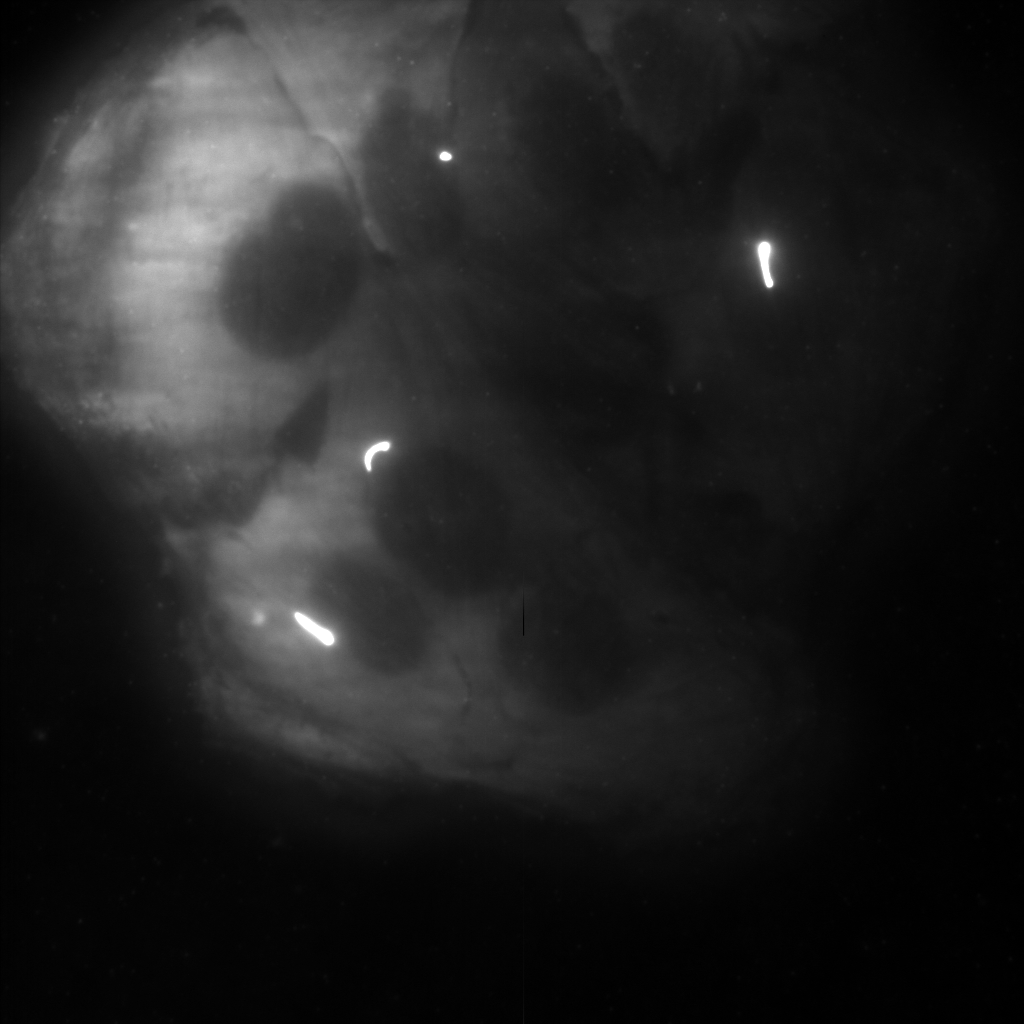

Supplement: Supplementary file 3 — Source Data Fig. 2 [file 44318_2024_60_MOESM3_ESM.zip › Figure 2/2D/WDR60 KO.tif]

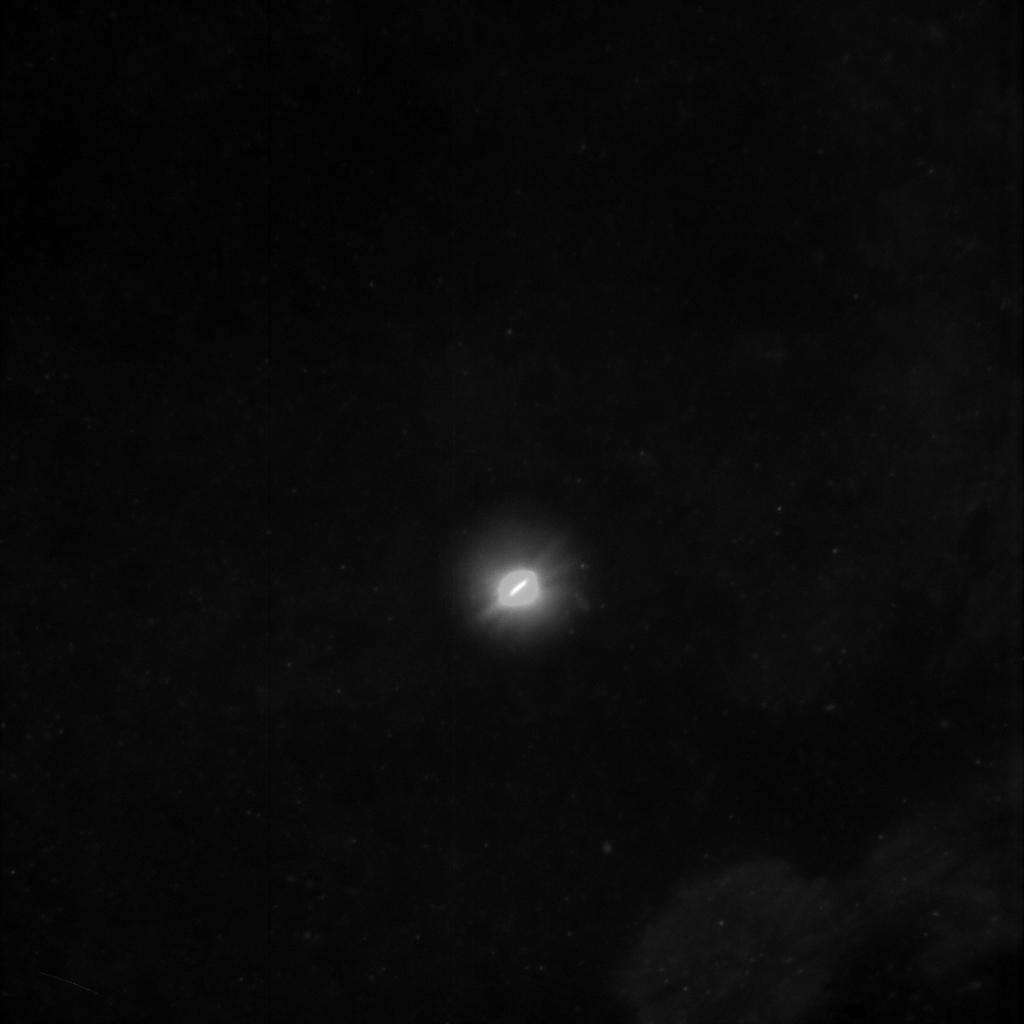

Supplement: Supplementary file 3 — Source Data Fig. 2 [file 44318_2024_60_MOESM3_ESM.zip › Figure 2/2D/Double KO.tif]

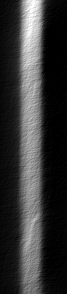

Supplement: Supplementary file 4 — Source Data Fig. 3 [file 44318_2024_60_MOESM4_ESM.zip › Figure 3/3B/Double KO Retrograde.tif]

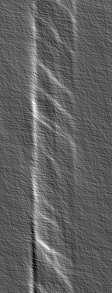

Supplement: Supplementary file 4 — Source Data Fig. 3 [file 44318_2024_60_MOESM4_ESM.zip › Figure 3/3B/WDR34 KO Anterograde.tif]

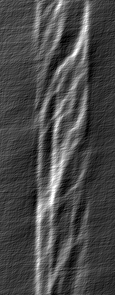

Supplement: Supplementary file 4 — Source Data Fig. 3 [file 44318_2024_60_MOESM4_ESM.zip › Figure 3/3B/Control Retrograde.tif]

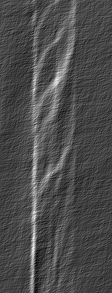

Supplement: Supplementary file 4 — Source Data Fig. 3 [file 44318_2024_60_MOESM4_ESM.zip › Figure 3/3B/WDR34 KO Retrograde.tif]

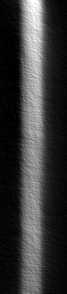

Supplement: Supplementary file 4 — Source Data Fig. 3 [file 44318_2024_60_MOESM4_ESM.zip › Figure 3/3B/Double KO Anterograde.tif]

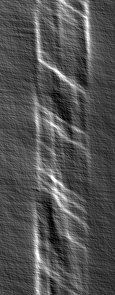

Supplement: Supplementary file 4 — Source Data Fig. 3 [file 44318_2024_60_MOESM4_ESM.zip › Figure 3/3B/Control Anterograde.tif]

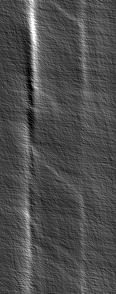

Supplement: Supplementary file 4 — Source Data Fig. 3 [file 44318_2024_60_MOESM4_ESM.zip › Figure 3/3B/WDR60 KO Anterograde.tif]

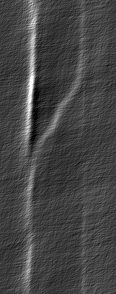

Supplement: Supplementary file 4 — Source Data Fig. 3 [file 44318_2024_60_MOESM4_ESM.zip › Figure 3/3B/WDR60 KO Retrograde.tif]

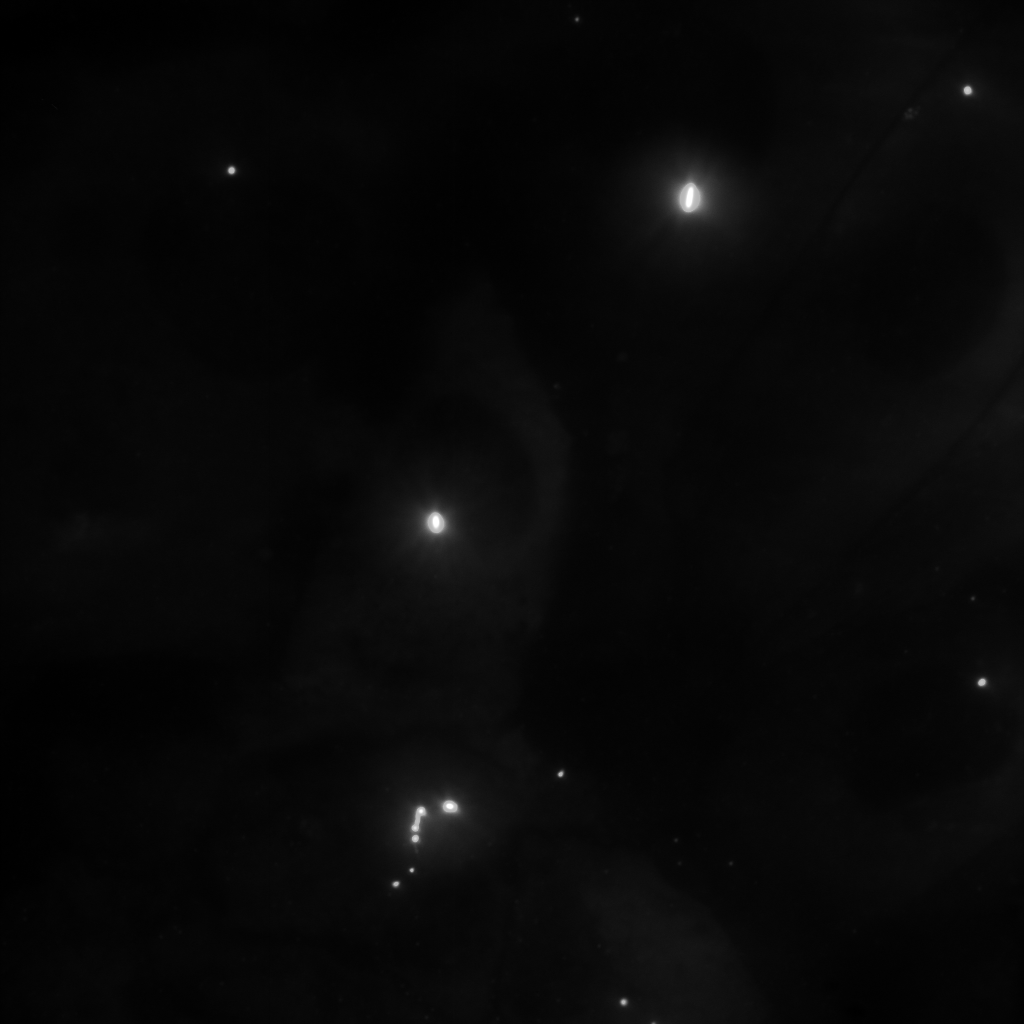

Supplement: Supplementary file 5 — Source Data Fig. 4 [file 44318_2024_60_MOESM5_ESM.zip › Figure 4/4A/Double KO NG3IFT88.tif]

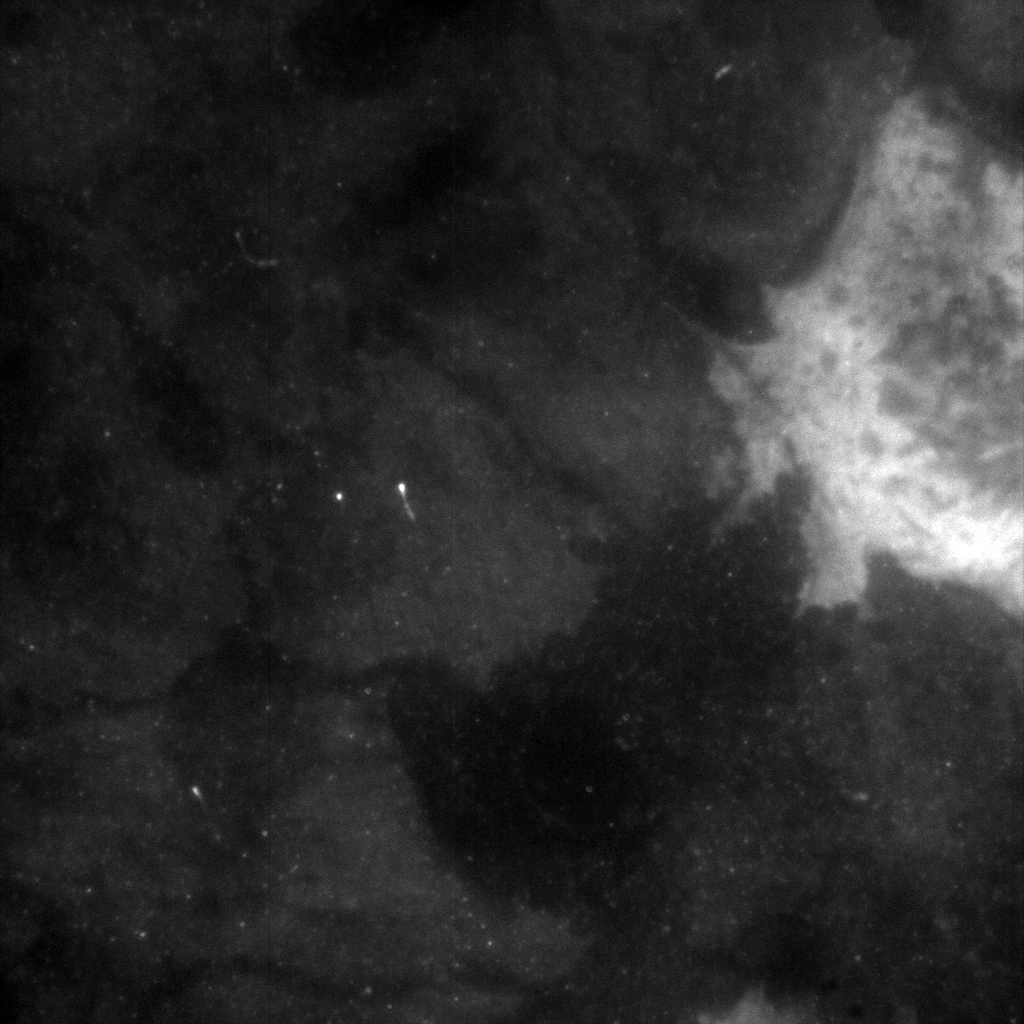

Supplement: Supplementary file 5 — Source Data Fig. 4 [file 44318_2024_60_MOESM5_ESM.zip › Figure 4/4A/WDR60 KO mScarDYNC2H1.tif]

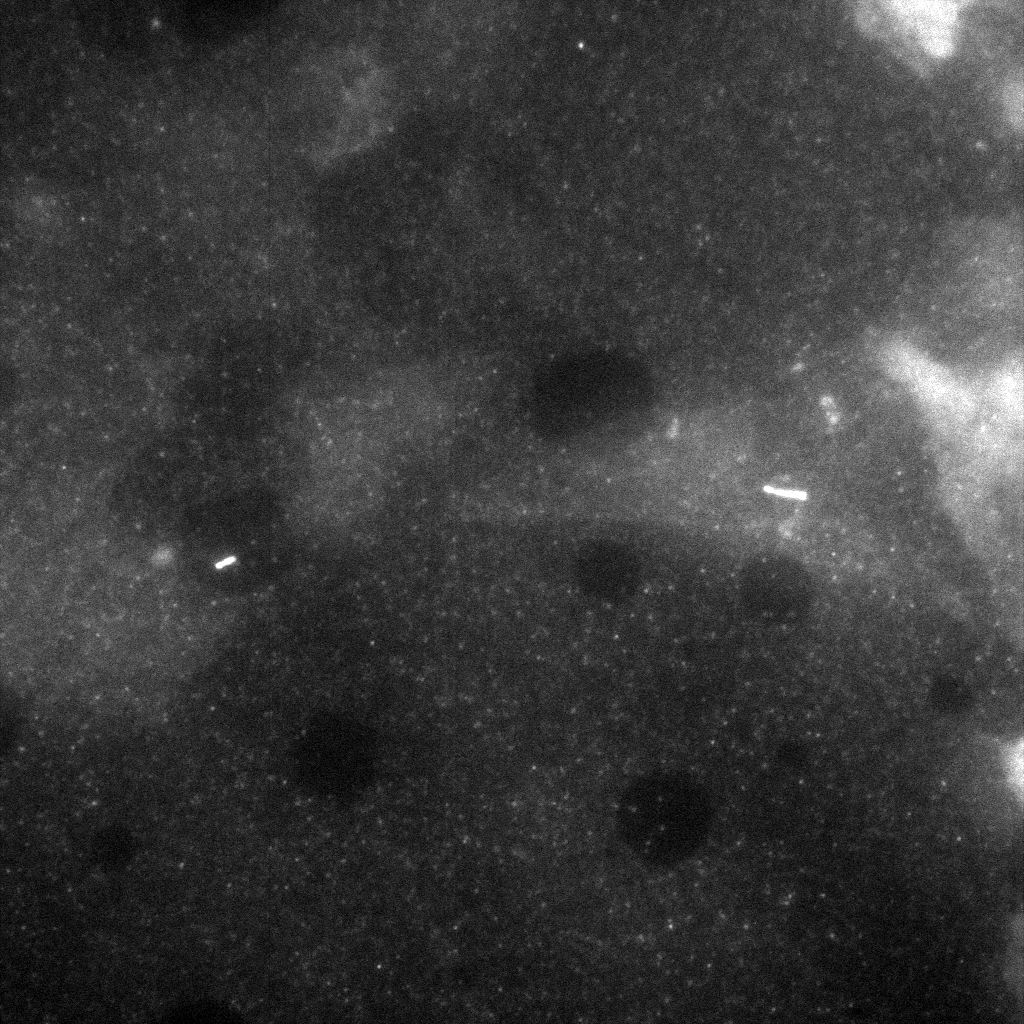

Supplement: Supplementary file 5 — Source Data Fig. 4 [file 44318_2024_60_MOESM5_ESM.zip › Figure 4/4A/WDR34 KO mScarDYNC2H1.tif]

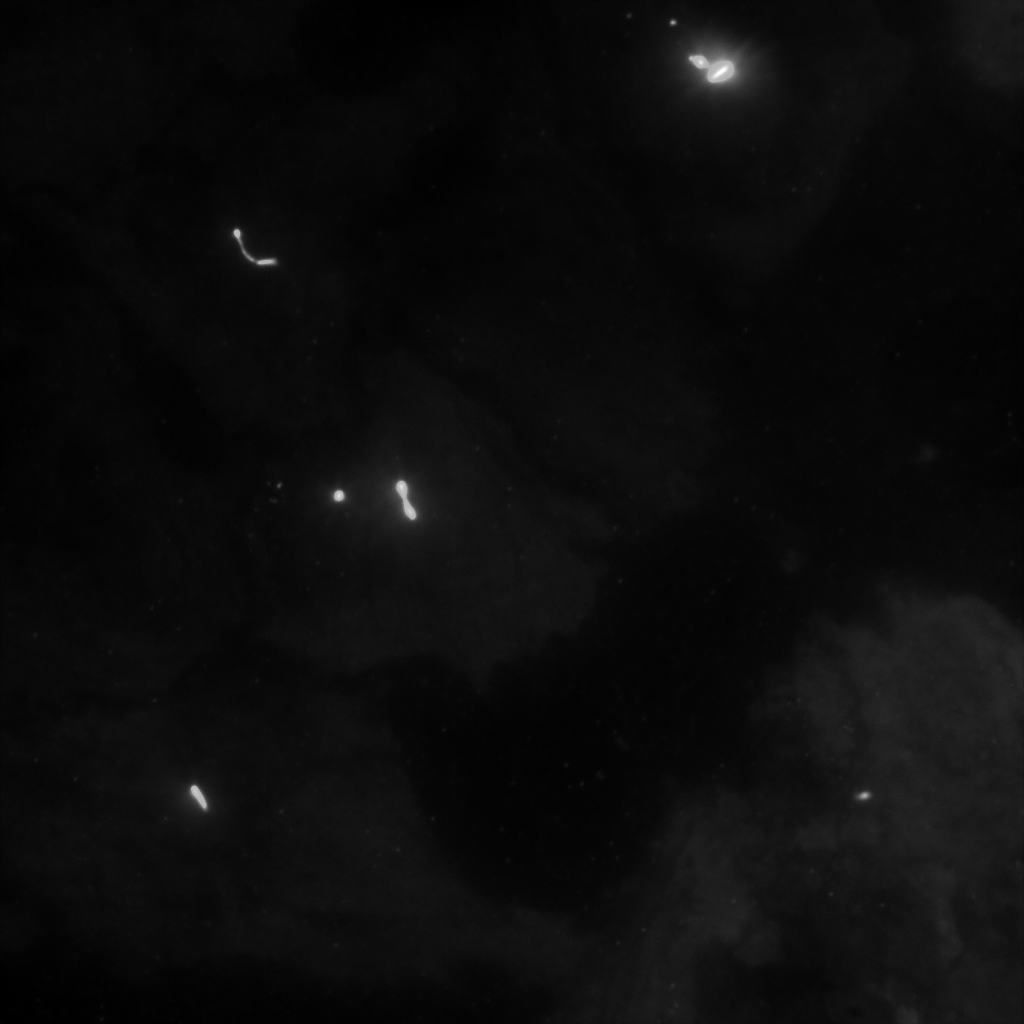

Supplement: Supplementary file 5 — Source Data Fig. 4 [file 44318_2024_60_MOESM5_ESM.zip › Figure 4/4A/WDR60 KO NG3IFT88.tif]

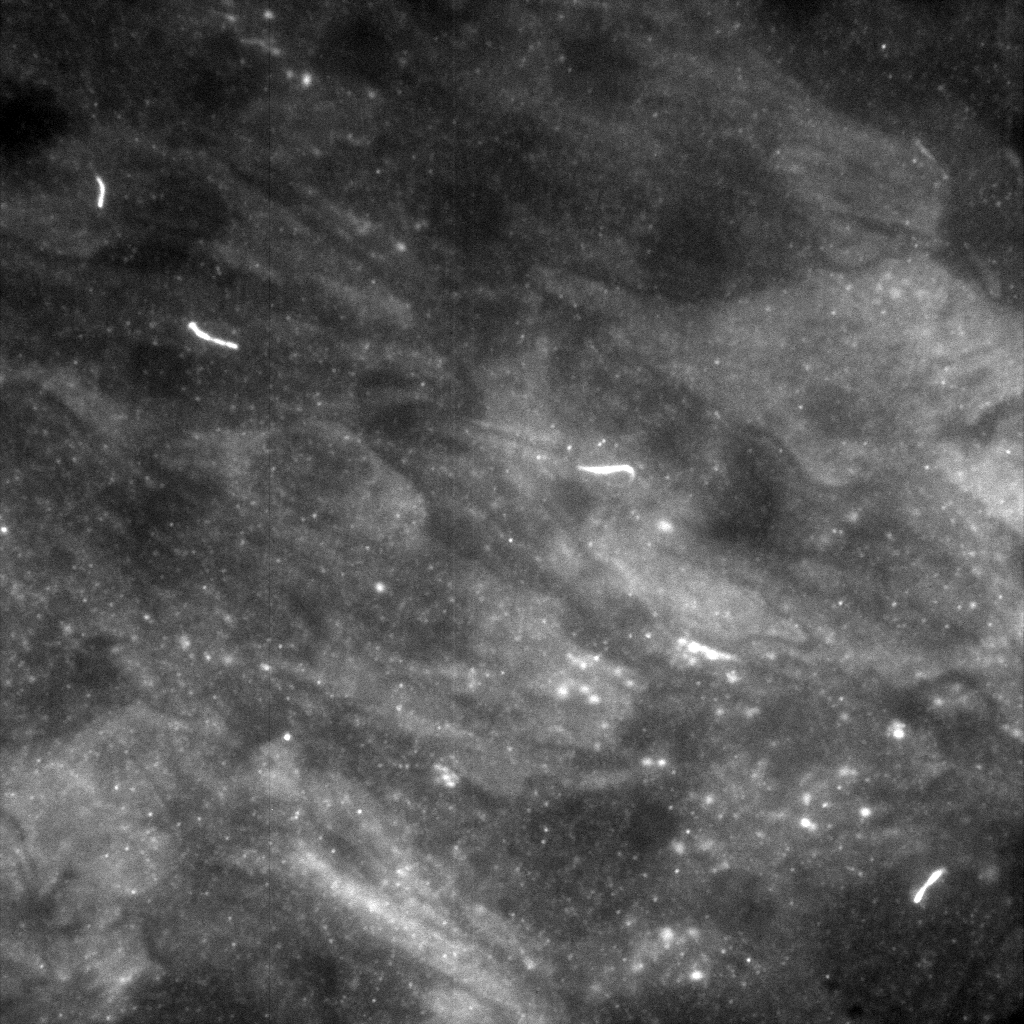

Supplement: Supplementary file 5 — Source Data Fig. 4 [file 44318_2024_60_MOESM5_ESM.zip › Figure 4/4A/Control mScarDYNC2H1.tif]

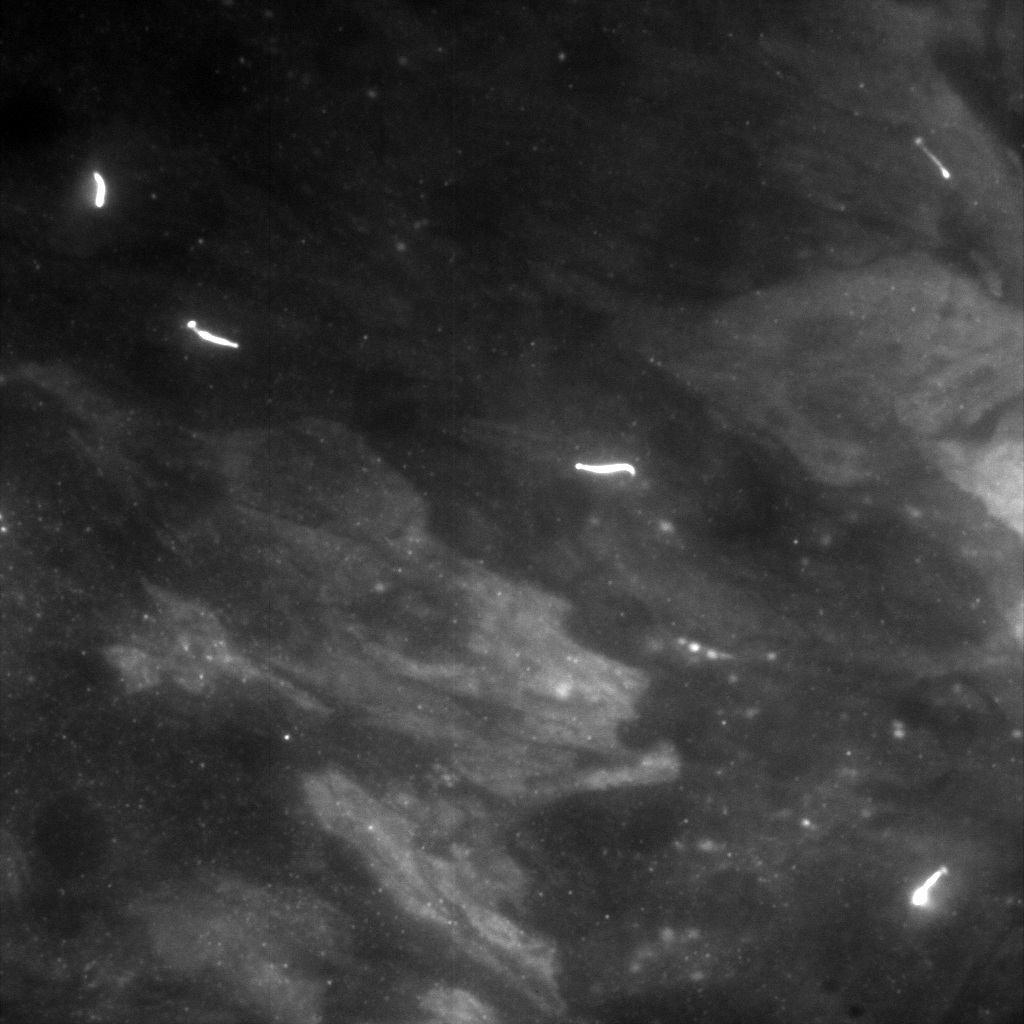

Supplement: Supplementary file 5 — Source Data Fig. 4 [file 44318_2024_60_MOESM5_ESM.zip › Figure 4/4A/Control NG3IFT88.tif]

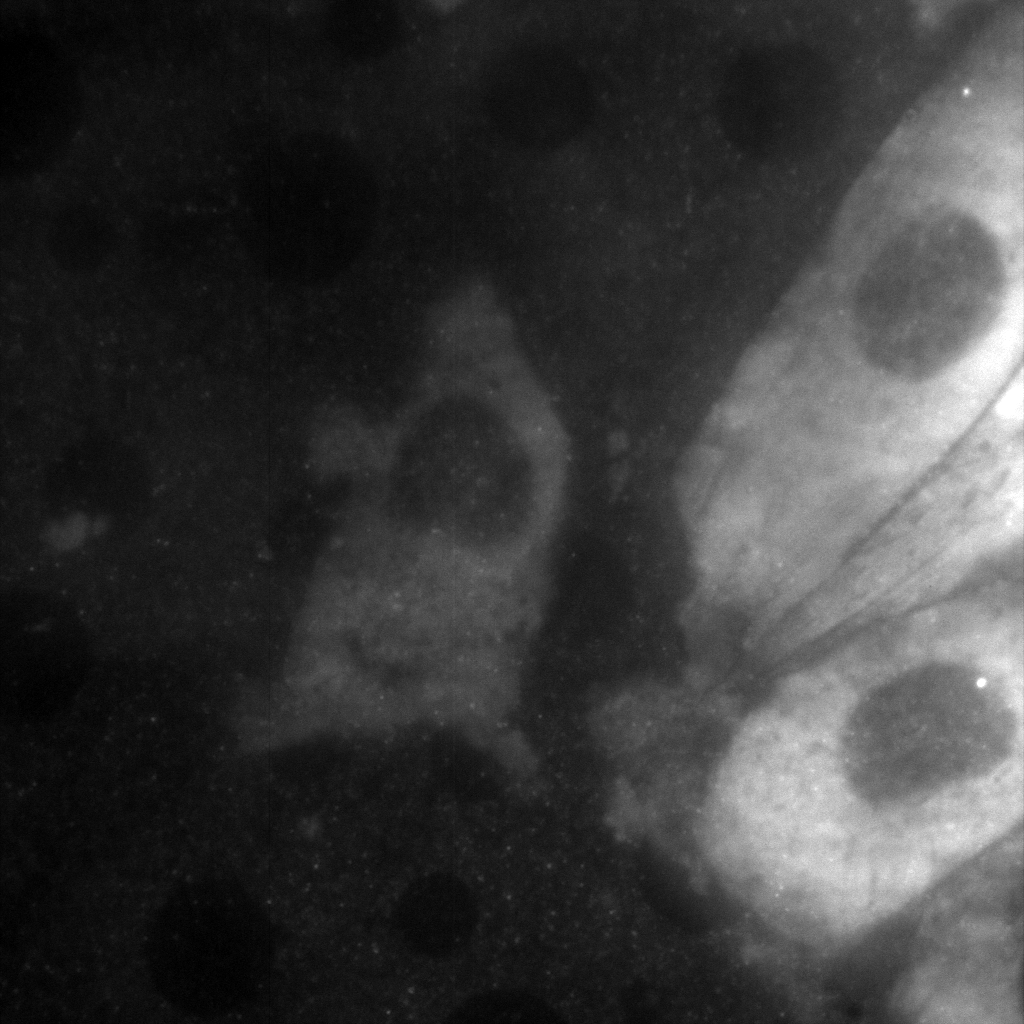

Supplement: Supplementary file 5 — Source Data Fig. 4 [file 44318_2024_60_MOESM5_ESM.zip › Figure 4/4A/Double KO mScarDYNC2H1.tif]

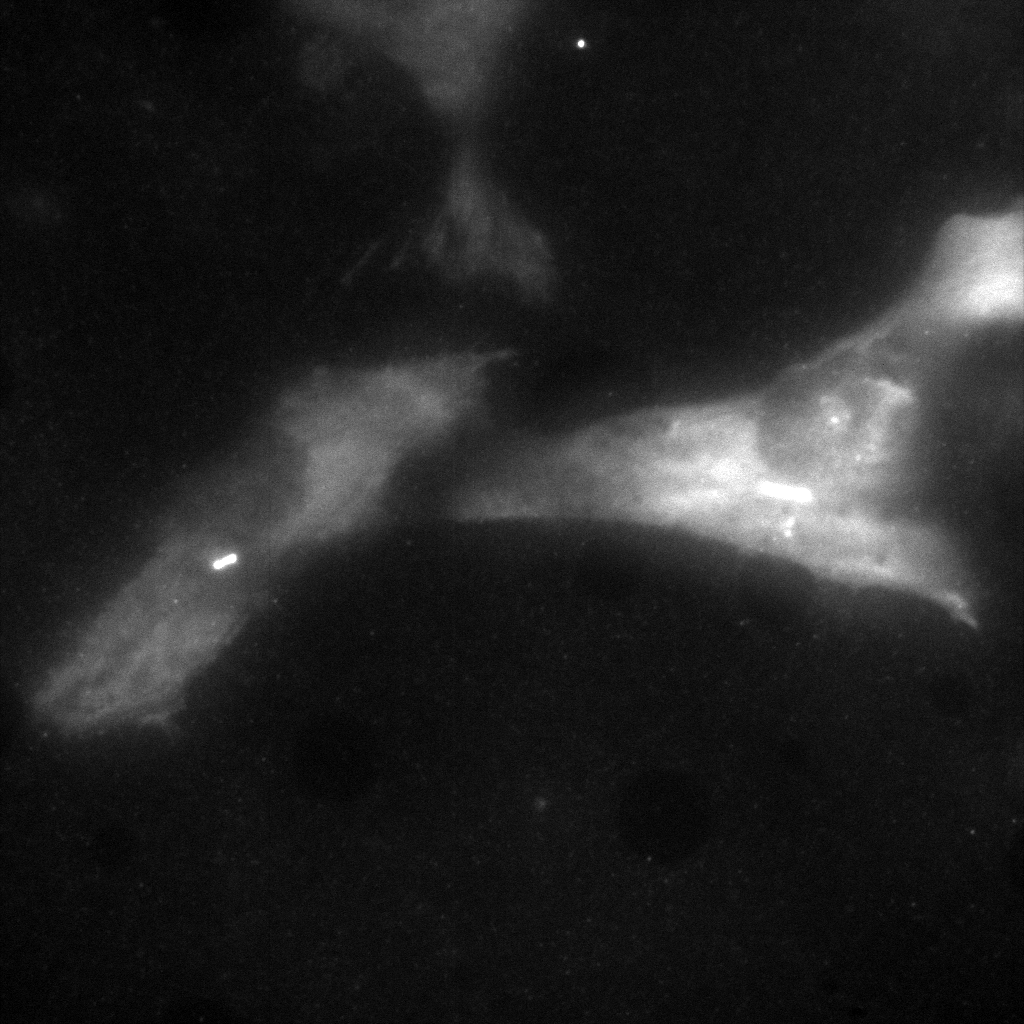

Supplement: Supplementary file 5 — Source Data Fig. 4 [file 44318_2024_60_MOESM5_ESM.zip › Figure 4/4A/WDR34 KO NG3IFT88.tif]

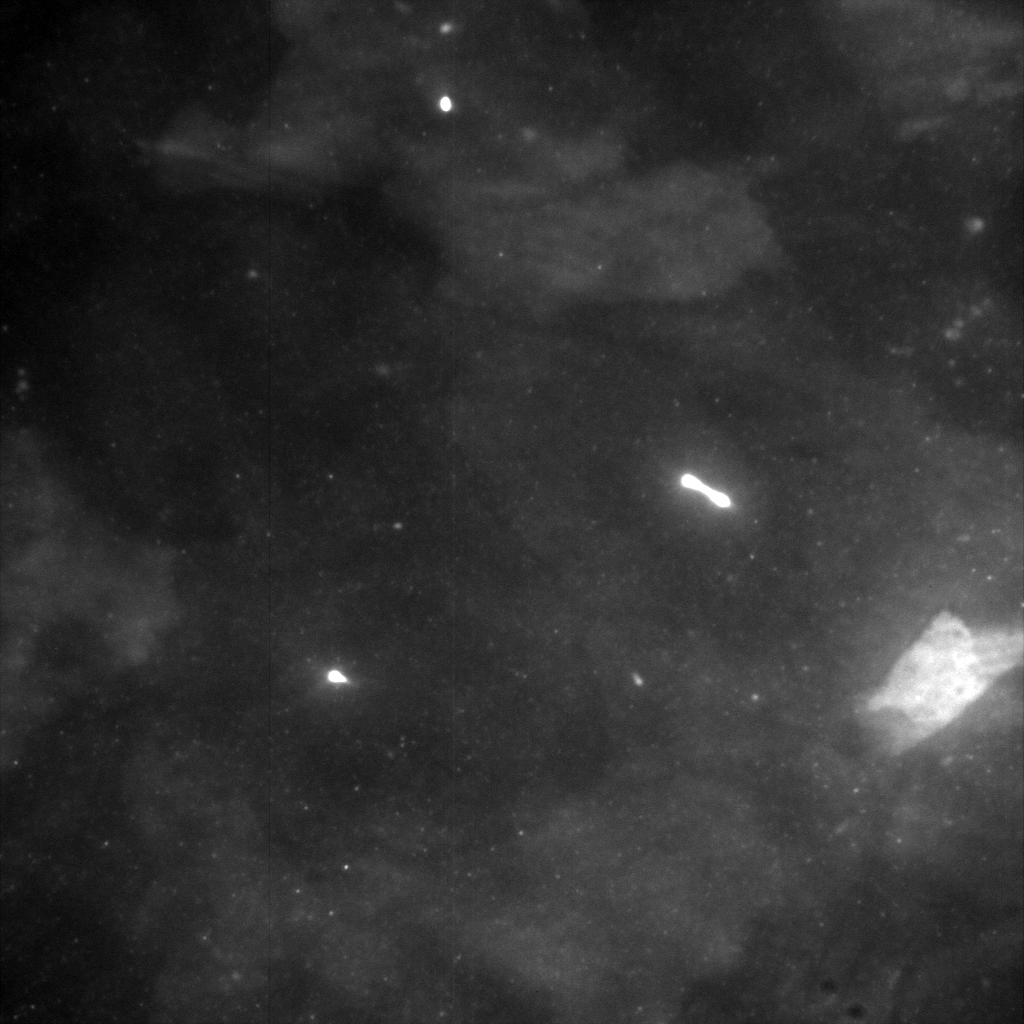

Supplement: Supplementary file 6 — Source Data Fig. 5 [file 44318_2024_60_MOESM6_ESM.zip › Figure 5/5H/WDR60_1-470_WDR34.tif]

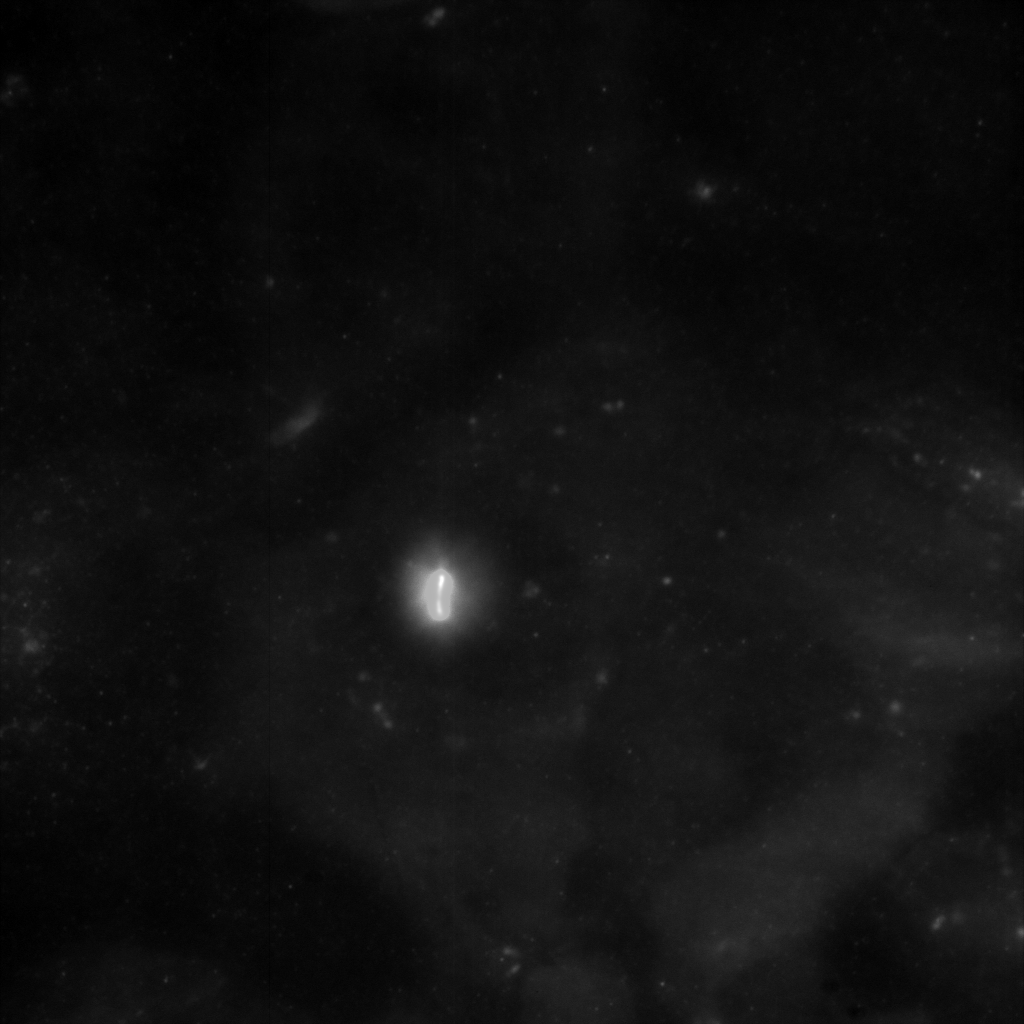

Supplement: Supplementary file 6 — Source Data Fig. 5 [file 44318_2024_60_MOESM6_ESM.zip › Figure 5/5H/WDR34WT.tif]

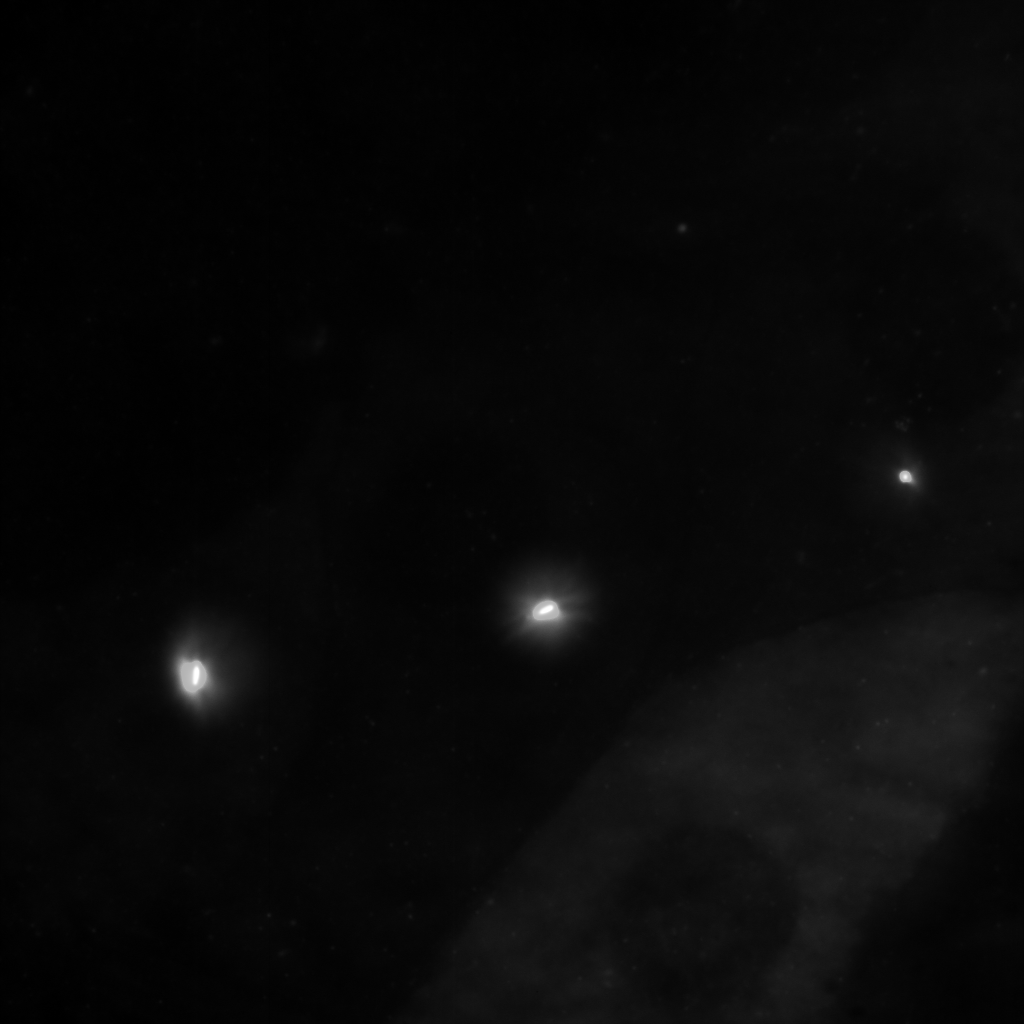

Supplement: Supplementary file 6 — Source Data Fig. 5 [file 44318_2024_60_MOESM6_ESM.zip › Figure 5/5H/Double KO.tif]

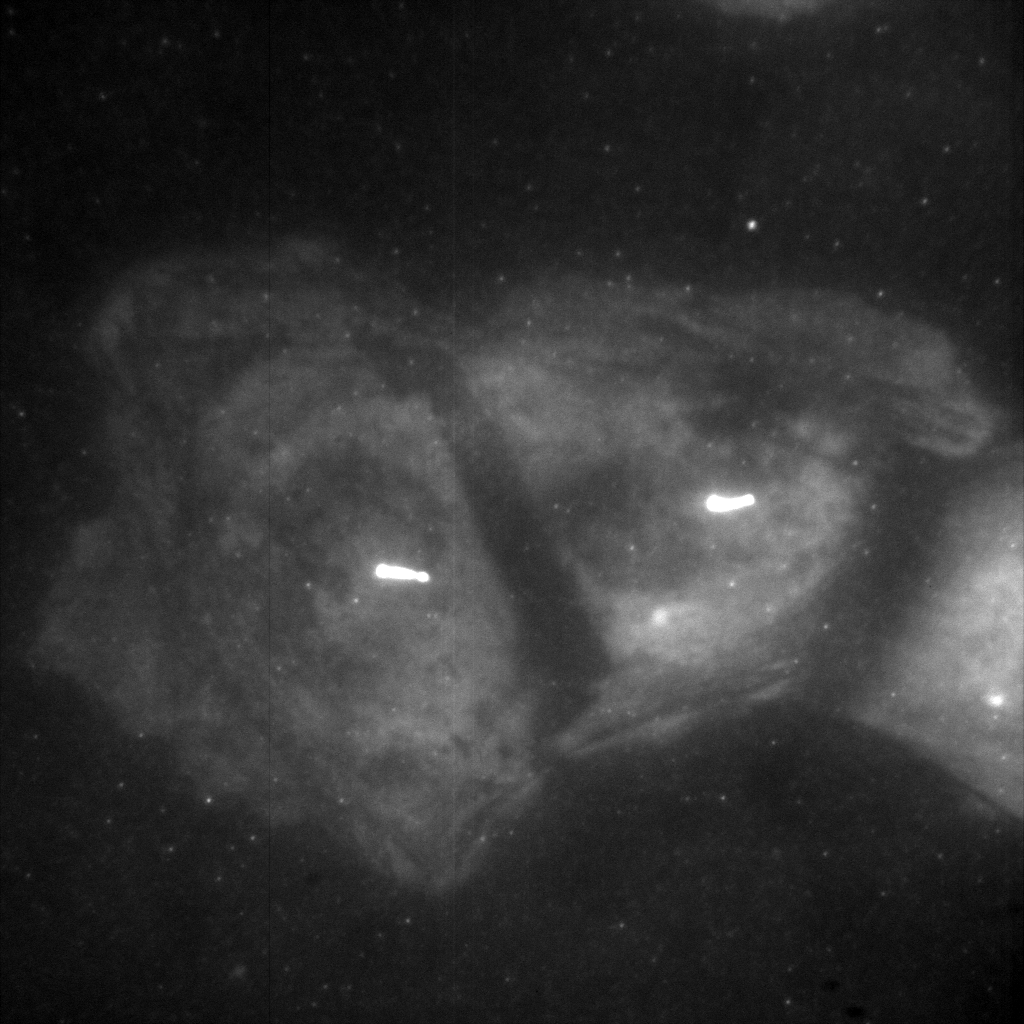

Supplement: Supplementary file 6 — Source Data Fig. 5 [file 44318_2024_60_MOESM6_ESM.zip › Figure 5/5H/WDR60WT.tif]

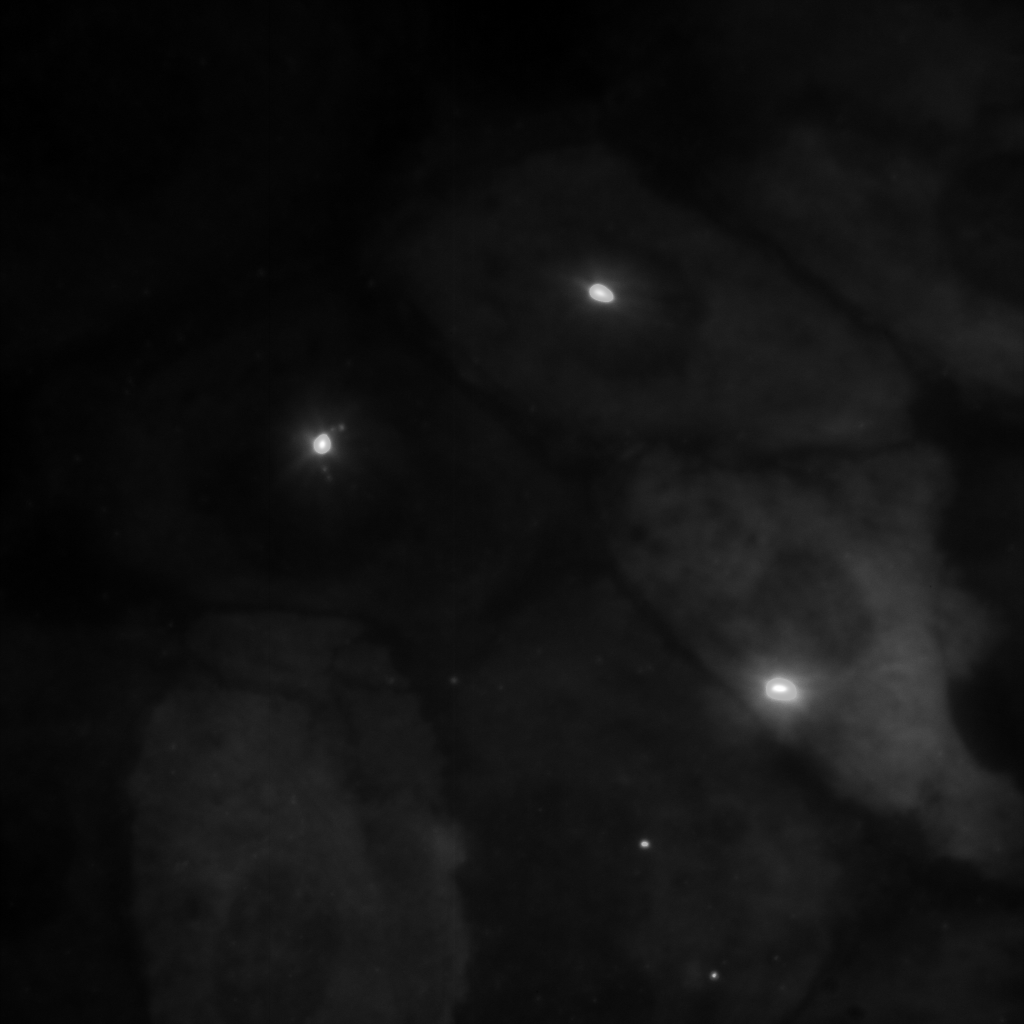

Supplement: Supplementary file 6 — Source Data Fig. 5 [file 44318_2024_60_MOESM6_ESM.zip › Figure 5/5E/dN470.tif]

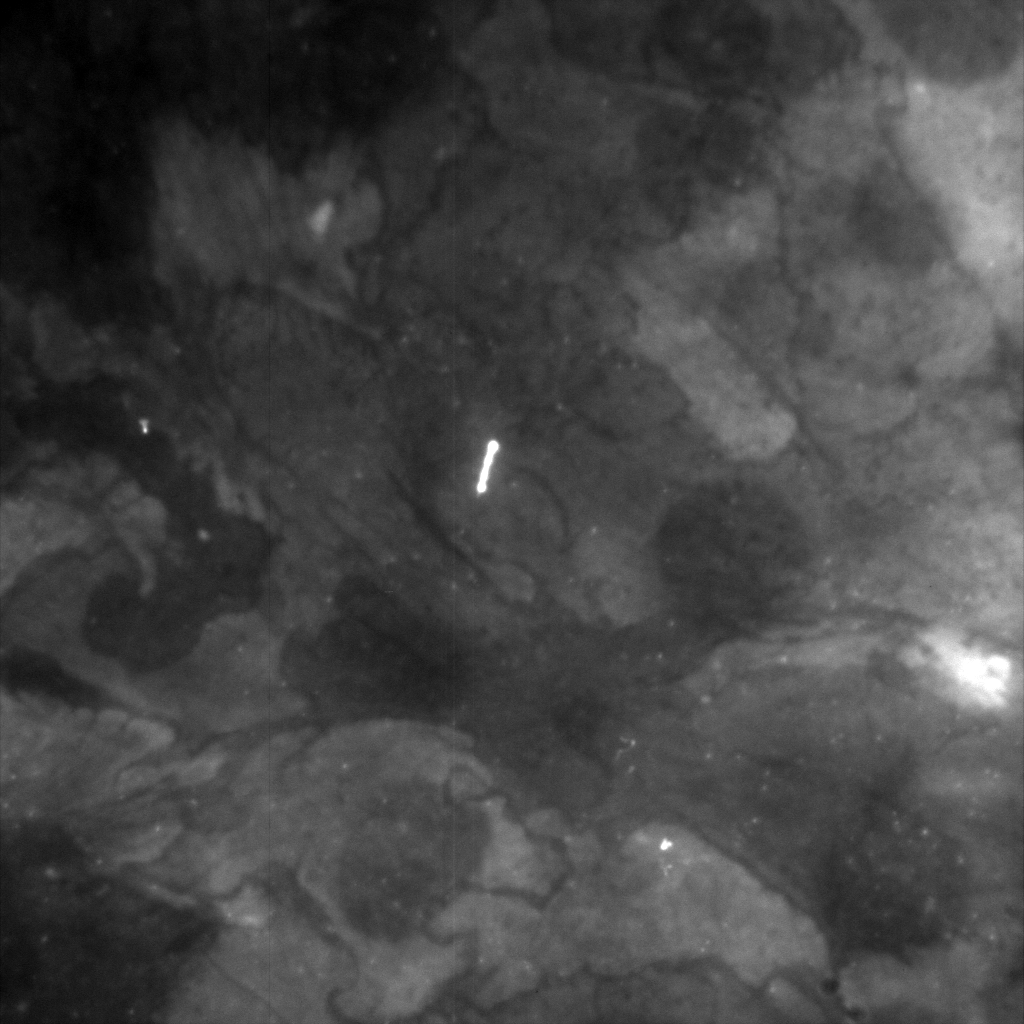

Supplement: Supplementary file 6 — Source Data Fig. 5 [file 44318_2024_60_MOESM6_ESM.zip › Figure 5/5E/WT.tif]

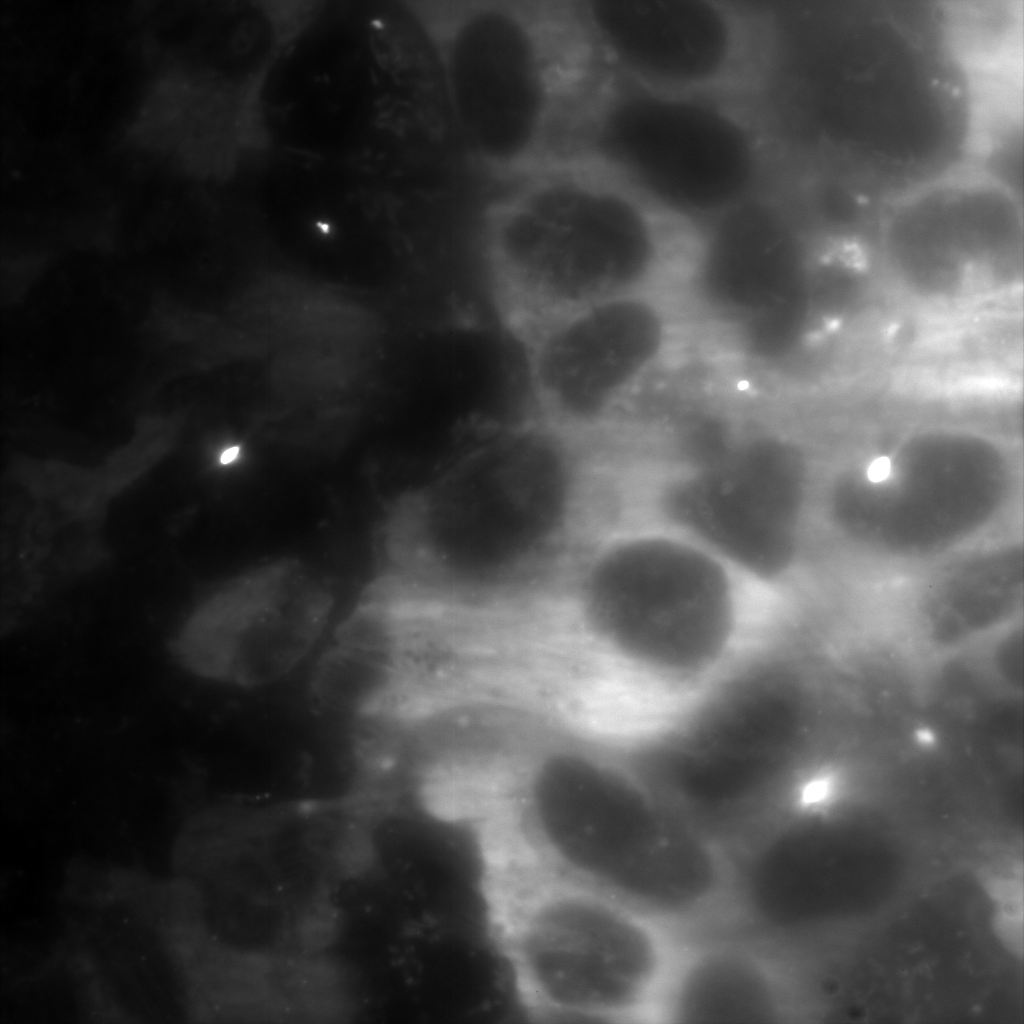

Supplement: Supplementary file 6 — Source Data Fig. 5 [file 44318_2024_60_MOESM6_ESM.zip › Figure 5/5E/Double KO.tif]

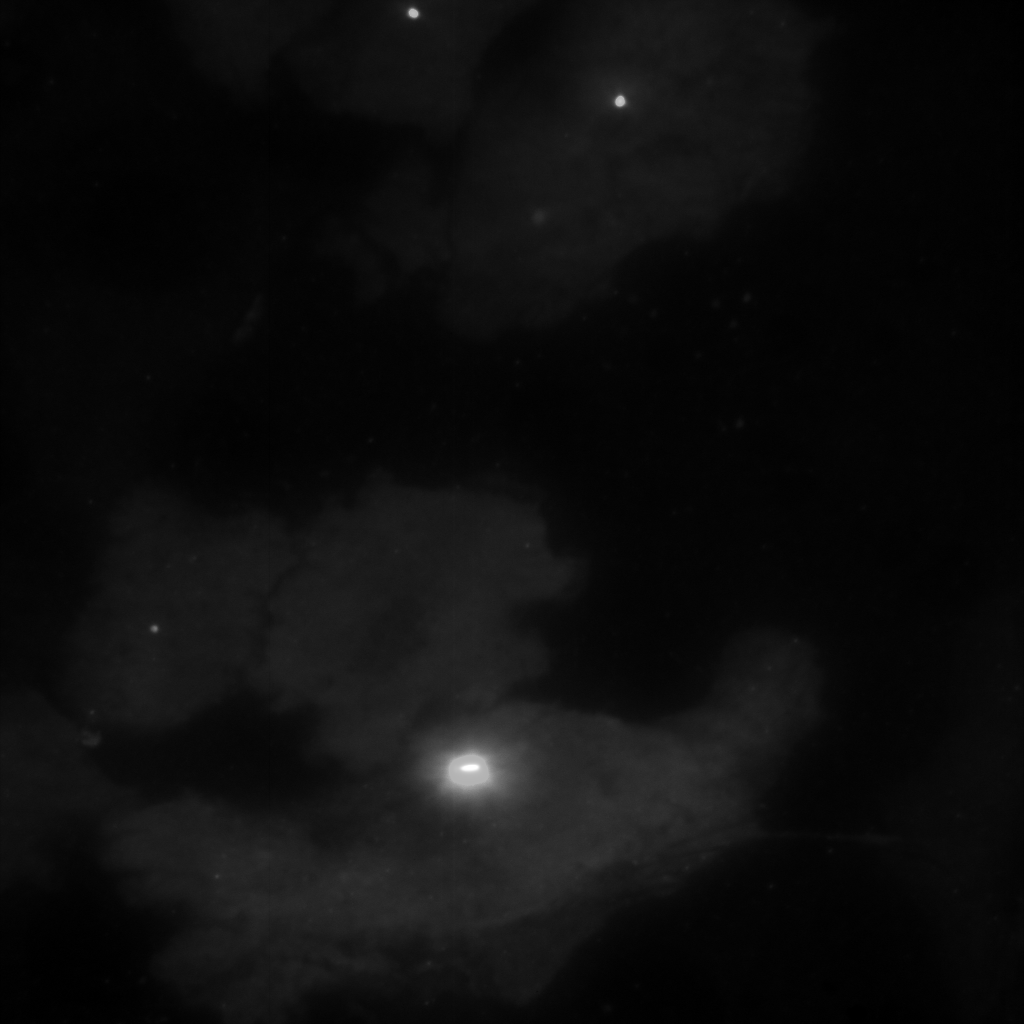

Supplement: Supplementary file 6 — Source Data Fig. 5 [file 44318_2024_60_MOESM6_ESM.zip › Figure 5/5E/dN630.tif]
